# The conserved noncoding RNA ModT coordinates growth and virulence in *Clostridioides difficile*

**DOI:** 10.1371/journal.pbio.3002948

**Published:** 2024-12-13

**Authors:** Tina Lenče, Johannes Sulzer, Kilian Andress, Anne-Sophie Gribling-Burrer, Vanessa Lamm-Schmidt, Lars Barquist, Redmond P. Smyth, Franziska Faber

**Affiliations:** 1 University of Würzburg, Faculty of Medicine, Institute of Molecular Infection Biology, Würzburg, Germany; 2 Helmholtz Institute for RNA-based Infection Research, Helmholtz Centre for Infection Research, Würzburg, Germany; 3 Université de Strasbourg, CNRS, Architecture et Réactivité de l'ARN, UPR9002, 67000 Strasbourg, France; 4 University of Würzburg, Faculty of Medicine, Würzburg, Germany; 5 Department of Biology, University of Toronto, Mississauga, Ontario, Canada; 6 University of Würzburg, Faculty of Medicine, Institute for Hygiene and Microbiology, Würzburg, Germany; Brigham and Women’s Hospital, UNITED STATES OF AMERICA

## Abstract

Bacterial noncoding RNAs fulfill a variety of cellular functions as catalysts, as scaffolds in protein complexes or as regulators of gene expression. They often exhibit complex tertiary structures that are a key determinant of their biochemical function. Here, we characterize the structured “*raiA* motif” RNA from *Clostridioides difficile*, which is conserved in more than 2,500 bacterial species from the phyla Bacillota and Actinomycetota. We show that its transcript abundance and stability in exponentially growing bacteria rivals that of ribosomal RNAs. Deletion of the “*raiA* motif” RNA is associated with delayed transition into stationary phase, and changes in stationary phase pathways such as spore formation, hence we rename it ModT (modulator of transition phase). Mechanistically, we show that ModT-mediated changes in cellular cyclic di-GMP levels are linked to the pronounced sporulation defect in the *modT* mutant. Importantly, we show that expression profiles and isoform patterns of ModT are conserved in *Clostridium perfringens* and *Paeniclostridium sordellii*, and that these orthologs can functionally complement ModT in *C*. *difficile*. Chemical structure probing of ModT *in vivo* reveals dynamic refolding and provides initial evidence for a potential association of ModT with proteins. In summary, our findings indicate that ModT fulfills a conserved role in regulating growth transitions in bacteria and provide a crucial step towards delineating its molecular mechanism.

## Abstract 2

Bacterial non-coding RNAs influence various cellular functions and often have complex tertiary structures. This study characterizes ModT, a conserved ncRNA found in 2,500 bacterial species, found to regulate growth transition and sporulation in Clostridioides difficile via changes in c-di-GMP.

## Introduction

Noncoding RNAs (ncRNAs) are central regulators of physiology, immunity and virulence in bacteria [1–3]. They act as ribozymes, scaffolds or regulators of gene expression, by providing fundamental housekeeping functions, defense against genetic invaders, and by optimizing gene expression in response to intracellular or environmental cues [4–6]. To serve this broad spectrum of functions, ncRNAs differ drastically in their sizes, structures, and molecular mechanisms.

The largest group of bacterial ncRNAs comprises small regulatory RNAs (sRNAs) that fine-tune gene expression at the posttranscriptional level. They often act in concert with RNA-binding proteins to regulate translation or mRNA stability, mostly through imperfect base pairing to their target mRNAs [7]. sRNAs typically display narrow conservation within a taxonomic species and are rarely found outside of their bacterial genus or family [8]. In comparison, ncRNAs with exceptional sequence or structural conservation are rare, and they fulfill conserved functions in all bacteria in which they are present. For example, tRNAs and rRNAs contribute to key steps of protein synthesis and are found in all forms of life. Other bacterial ncRNA families with deep structure and/or sequence conservation include 6S RNA, SsrA, the RNA of the Signal Recognition Particle or RNaseP. They all accomplish unique biological functions that are conserved across all bacteria [9–14]. Beyond these few well-known ncRNA families, computational screens of intergenic microbial metagenome sequences have revealed a range of motifs with deep primary sequence and secondary structure conservation [15,16]. While many of them are likely to be *cis*-acting riboswitches, the size, structural complexity, and genomic location of some motifs make them candidates for large structured RNAs with potential catalytic roles, such as ribozymes [17,18]. Therefore, their characterization holds great promise to reveal novel RNA functions. This is exemplified by the Ornate Large Extremophilic (OLE) RNA, which is encoded in many extremophilic gram-positive bacteria and supports the response to diverse types of stressors in *Bacillus halodurans*, at least in part by forming a membrane-bound, stress-responsive ribonucleoprotein particle with the OapA protein [19,20].

Using RNA sequencing-based annotation of transcript start sites and transcript ends in the spore-forming anaerobe *Clostridioides difficile* (*C*. *difficile*), we recently characterized the ncRNA landscape in this important nosocomial pathogen [21]. This analysis highlighted expression of an RNA, termed “*raiA* motif” RNA (RF03072), which is a member of a largely uncharacterized RNA family. Based on our results described below, we have renamed this RNA ModT, for modulator of transition phase (TP). ModT can be found in more than 2,500 biologically diverse species, mostly belonging to the phyla Bacillota and Actinomycetota [15,16]. Intriguingly, a recent study in *Clostridium acetobutylicum* demonstrated that deletion of the *modT* locus has a negative impact on spore formation and bacterial aggregation [22]. Sporulation is a wide-spread developmental process in bacteria that allows long-term survival under adverse conditions. In *C*. *difficile*, which is the leading cause of healthcare-associated infective diarrhea, spore formation is crucial for host transmission and survival of antibiotic treatment in infected patients [23–25]. Hence, understanding the regulatory principles underpinning this process is crucial to devise much needed therapeutic interventions in the future.

However, the presence of ModT in many nonspore-forming bacteria strongly suggests that it regulates a more fundamental process that is conserved across these biologically diverse *modT-*encoding bacteria. This hypothesis is further supported by the highly conserved and complex tertiary structure of ModT, that is reminiscent of ribozymes. To gain first insights into potential functions of ModT, Soares and colleagues performed a comprehensive analysis of its gene synteny [22]. Since genes that participate in the same cellular process often co-localize in bacterial genomes, it might indicate potential biochemical and biological functions of ModT. The analysis revealed that *comFC* genes (74%) and *raiA* genes (66%) occur most commonly up- or downstream of *modT*, respectively. In *C*. *difficile*, however, the downstream gene is encoding a transcription antiterminator of a PTS operon instead of *raiA*. In addition to differences in neighboring gene functions, there is variability in the orientation and distance of *modT* to its neighbor genes across bacteria. Therefore, gene synteny does not appear to be a good indicator of potential cellular functions of ModT.

Here, we perform an in vivo characterization of ModT in *C*. *difficile*. Our study identifies ModT as an abundant and highly stable *trans-*acting RNA that impacts the transition from the exponential to the stationary growth phase. This regulatory function of ModT has direct impact on the expression of stationary phase pathways such as sporulation and toxin production, via changes in cellular c-di-GMP levels. We further show that *C*. *difficile* ModT can be functionally replaced by several distant orthologs with conserved core structural features. Our work provides functional evidence that ModT fulfills a conserved cellular function that is likely enabled by its highly preserved tertiary structure.

## Results

### ModT transcript abundance and stability resembles housekeeping RNAs

**Fig 1:**
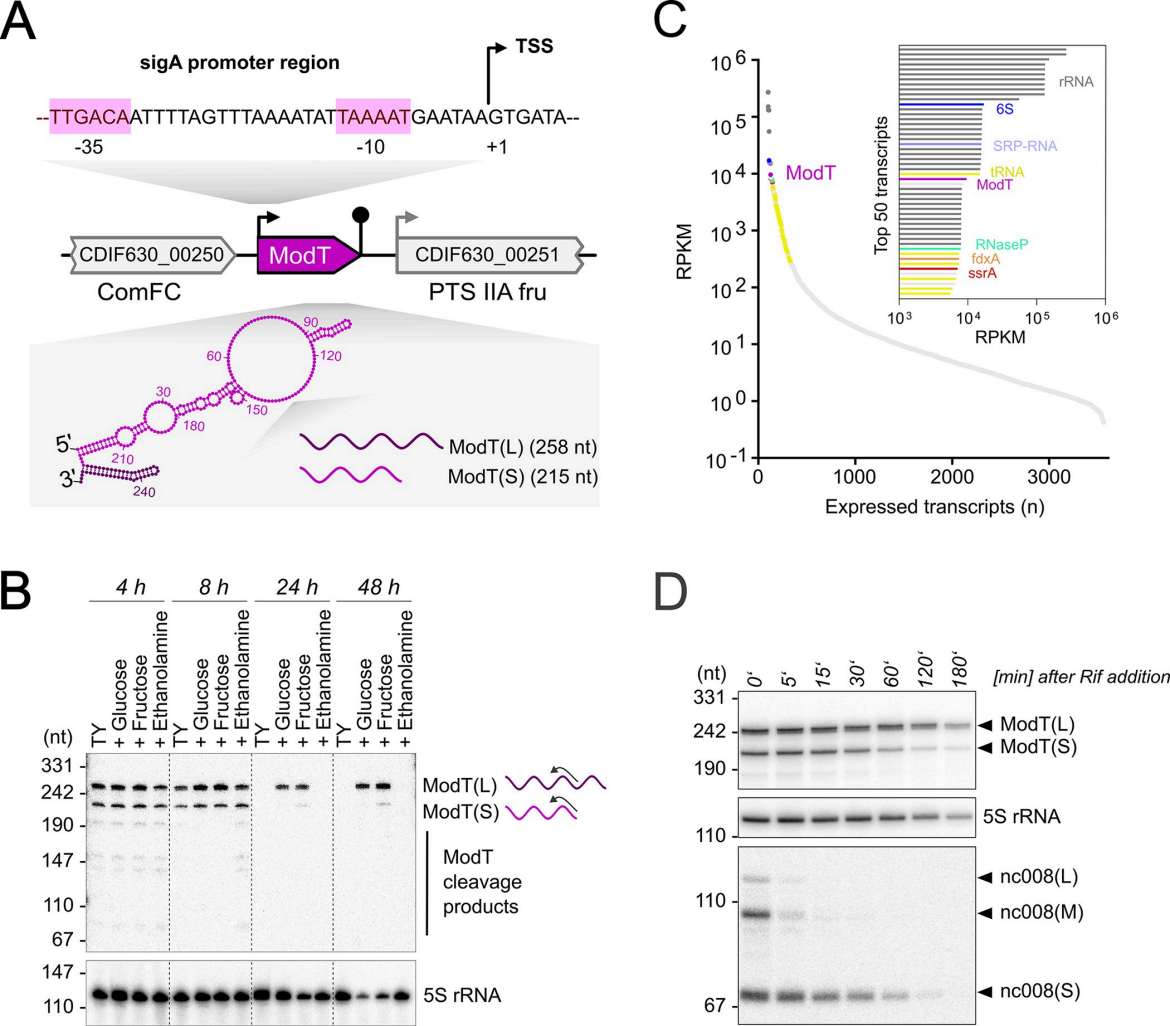
ModT is a highly abundant and stable noncoding RNA. ** (A)** Schematic of the *modT* locus in *C*. *difficile* 630. Promoter region including the SigA -35 box and -10 box are shown on top, TSS and TTS are indicated with an arrow or a pin symbol, respectively. In silico secondary structure prediction (RNAfold) of the *modT* RNA is displayed in pink, with the 3′-end extension of the long isoform highlighted in purple. **(B)** ModT expression profile in *C*. *difficile* 630 across different growth stages. Total RNA was extracted from *C*. *difficile* 630 grown in TY medium supplemented with indicated carbon sources at indicated time points (for reference, see growth curves shown in Fig 3B). ModT transcript was detected by northern blot using a radioactively labeled DNA probe (FFO-67) that captured both ModT isoforms, as indicated with an arrow. 5S rRNA served as loading control. **(C)** Normalized read distribution of all expressed and annotated transcripts (*n* = 3,472) in *C*. *difficile* 630 sorted by transcript abundance [26]. Inset displays the 50 most abundant transcripts, with ModT labeled in pink. The underlying data can be found in S1 Dataset. **(D)** In vivo RNA half-lives for different noncoding RNA transcripts. *C*. *difficile* 630 was grown to mid-exponential phase and treated with Rifampicin. Total RNA was extracted at the indicated time points and analyzed by northern blotting using radioactively labeled DNA probes. The representative of 3 independent experiments is shown. TSS, transcriptional start site; TTS, transcription termination site.

We previously demonstrated expression of ModT in *C*. *difficile* through genome-wide RNA sequencing-based mapping of transcriptional start sites (TSS) and transcript ends (annotated CDIF630nc_001 in [21,26]). ModT caught our attention due to its high sequence and secondary structure conservation across 9 taxonomic classes and 2 bacterial phyla [22]. RNA-seq-based transcript annotation revealed that expression of ModT is driven from a single intergenic TSS that is associated with a canonical housekeeping σ^A^ (SigA) promoter (**Figs 1A and S1A** [21]). Transcription of *modT* gives rise to a long (ModT (L), 260 nt) and a short (ModT(S), 215 nt) isoform that differ in their 3′ ends (**Figs 1B and S1B**). Again, our previous RNA-seq-based transcript annotation indicates 2 independent transcription termination events at sites that correspond to transcript lengths detected by northern blot (**S1A Fig**). Interestingly, analysis of existing RNA-seq data [26] shows that ModT is among the 30 most abundant transcripts in the cell, and only surpassed by some, but not all, ribosomal RNAs and tRNAs, as well as 6S RNA and the signal recognition particle RNA (**Fig 1C** and **S1 Dataset**).

To develop a better understanding of ModT function, we initially performed northern blot analysis to characterize its expression across growth stages and in the presence of different carbon and energy sources (**Figs 1B** and **S1B**). We observed prominent expression of both isoforms in TY (Tryptone-Yeast) medium throughout exponential growth, but it decreased to the limit of detection after transition into the stationary phase. In contrast, during growth on monosaccharides, such as glucose or fructose, steady-state levels of ModT(L), but not ModT(S) remained high even in late stationary phase. Since both isoforms are transcribed from the same promoter, we sought to explore whether their different fates in the stationary phase are determined by different RNA half-lives. Therefore, we monitored RNA levels over time, after Rifampicin-mediated inhibition of de novo *modT* transcription (**Figs 1D** and **S1C**). Intriguingly, the long isoform was substantially more stable (t_1/2_ = >180 min) than the short isoform (t_1/2_ = 45.94 min). This suggests that the accumulation of the long isoform in the stationary phase is, at least in part, due to a longer half-life relative to the short isoform. Moreover, the ModT(L) half-life was similar to the housekeeping 5S rRNA (t_1/2_ = >180 min) and exceeded by far that of the canonical Hfq-dependent small RNAs CDIF630nc_008 (t_1/2_ = 22.95 min) (**Figs 1D** and **S1C**). In addition, we also observed a higher GC content of ModT (45.6% for ModT(S) and 42.6% for ModT(L)) compared to the average GC content of the *C*. *difficile* genome (29.1%) or previously annotated sRNAs (27.5%) (**S1D Fig**). In summary, the ModT transcript exhibits several features that are reminiscent of housekeeping RNAs and suggests that this noncoding RNA fulfills a fundamental physiological function.

### ModT isoforms adopt identical structures in vitro and in vivo

**Fig 2:**
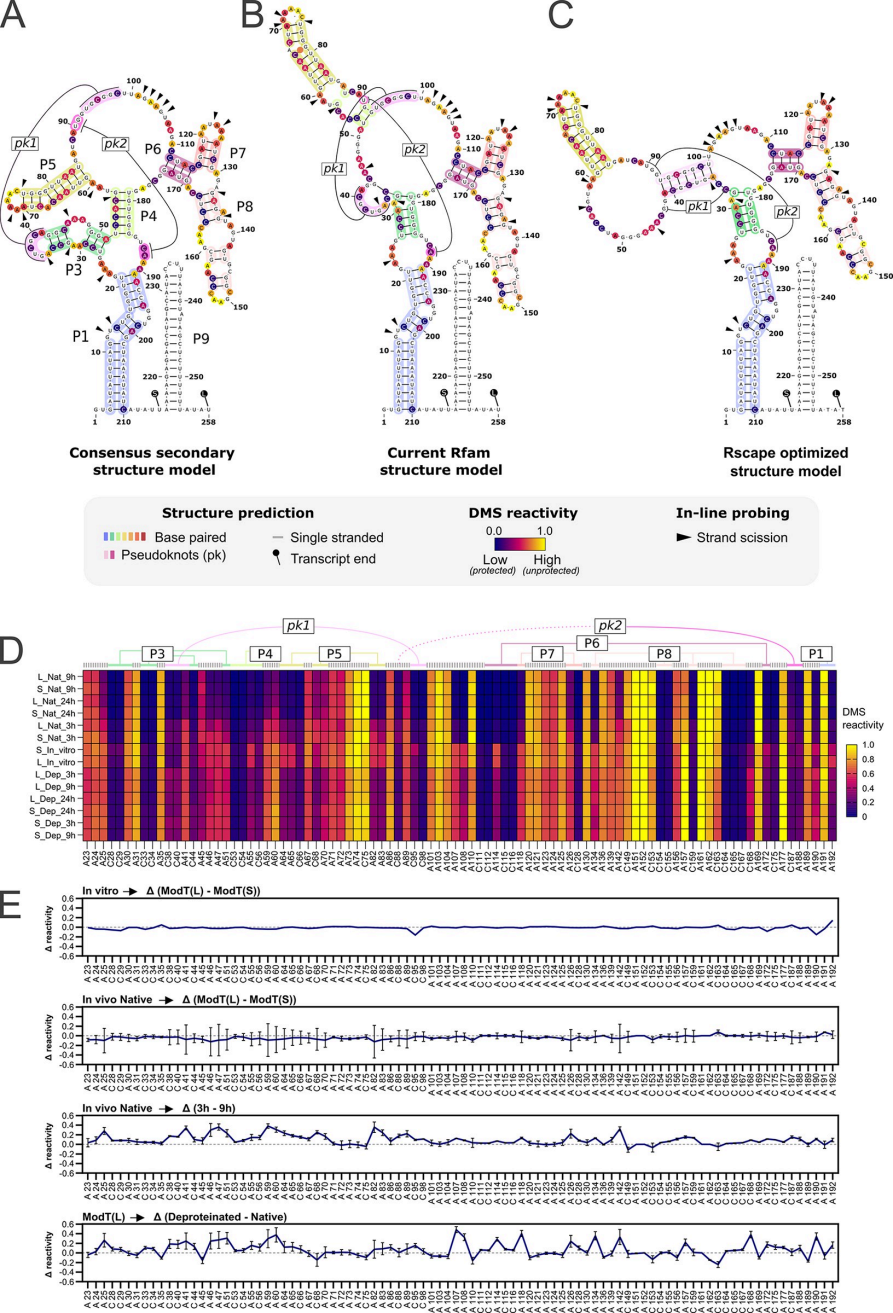
DMS probing uncovers structural dynamics of ModT during different growth phases and test conditions. **(A–C)** Manually curated secondary structure models of *C*. *difficile* 630 ModT based on available Rfam models (RF3072). The stem loop structure of the appended 3′ end is based on predictions by RNAfold. (A) Consensus secondary structure as described in [15,22], (B) current Rfam structure, and (C) the R-scape optimized structure of *C*. *difficile* 630 ModT. Black triangles indicate the most prominent scission events determined by In-line probing (see S2A–S2D Fig). Average DMS-reactivity for each residue, derived from in vitro structure probing of ModT(S) and ModT(L), is coded with a color-gradient (filled circles). **(D)** DMS reactivity profiles of adenine (A) and cytosine (C) nucleotides for residues 19–210 of ModT in following conditions: in vitro, in vivo native (Nat), and in deproteinated cell lysates (Dep). The Nat and Dep samples were analyzed in EP (3 h), TP (9 h), and stationary phase (24 h) of growth. Under “Nat” and “Dep” conditions, L displays reactivity specific for the long isoform, while S represents combined reactivities of the short and the long RNA isoforms. The conditions are sorted according to sample clustering in S2E Fig. The dendrogram has been omitted for clarity. The underlying data can be found in S5 Dataset. **(E)** Comparisons of DMS reactivity in different conditions or isoforms. The Δ reactivity for each nucleotide position was calculated by subtraction of normalized DMS reactivities obtained for the indicated conditions (see top of each graph). For “in vivo Native → Δ(ModT(L)—ModT(S))” and “ModT(L) → Δ(Deproteinated–Native),” the mean subtracted reactivity and its standard deviation was calculated from all growth stages (3 h, 9 h, and 24 h). For “In vivo Native → Δ(3h - 9h),” the profiles of ModT(L) and ModT(S) were combined to obtain the mean reactivity and standard deviation at these 2 time points. The underlying data can be found in S5 Dataset. EP, exponential phase; TP, transition phase.

The function of many RNA elements is determined by their unique structure [27]. Given that both ModT isoforms exhibit very distinct in vivo half-lives, we wanted to explore whether they fold into distinct structures. First, we compared the *C*. *difficile* ModT sequence with 3 structure models for ModT, including the original structure predicted for ModT [15,22], the structure available from the Rfam database (RF03072), and a predicted structure optimized with R-scape (**Fig 2A–2C**). Of note, all 3 models represent the short isoform, likely because sequence conservation is poor in the extended 3′-end [15], and we appended our predicted terminator sequence to them. The recently updated consensus secondary structure model for ModT [22] has 2 pseudoknots (denoted pk1 and pk2) and 8 predicted base-paired regions (denoted P1 through P8), and except for P2, all of them are present in both isoforms of *C*. *difficile* ModT (**Fig 2A**). We also mapped the *C*. *difficile* ModT sequence onto the current Rfam structure (**Fig 2B**) as well as the R-scape optimized structure (**Fig 2C**) [28,29]. The 3 models show prominent structural differences in the regions comprising P3 and P4, as well as in the residues involved in potential pseudoknot formations. Therefore, we decided to include all of them in our analysis.

In-line probing assays (ILP) on in vitro transcribed ModT(L) and ModT(S) (**Figs 2A–2C and S2A–S2D**) provided general support for all 3 in silico predicted structure models. More specifically, ILP indicated prominent cleavage events at nucleotides mostly predicted to be unpaired in each model. Notably, ILP of ModT(L) resulted in a cleavage pattern identical to ModT(S), suggesting that the extended 3′-end adopts a fold that does not alter the core structure of ModT(L). However, given that the structural information resolved by the ILP is limited for larger RNAs, we decided to obtain better insight into the ModT structure both in vitro and in vivo using dimethyl sulfate mutational profiling (DMS-MaP) [30,31]. DMS-MaP is a well-established method to infer secondary structure by exploiting DMS reactivity at single stranded, or exposed, A and C nucleotides. We generated DMS-reactivity profiles of ModT(S) and ModT(L) under various conditions: (1) in vitro transcribed ModT isoforms; (2) in vivo, from cultures grown to exponential, transition, and stationary phase; and (3) from deproteinated cell lysates obtained at the same growth phases (**Fig 2D**). Of note, we specifically determined the reactivity values of the long isoform (L) using a primer binding to the extended 3′-end for reverse transcription in vivo and in deproteinated cell lysates. As this is not possible for the short isoform, reactivities of these samples (S) represent a combined contribution of ModT(S) and ModT(L), which might mask minor structural differences.

Importantly, DMS reactivities for both ModT isoforms showed very high correlations (≥0.98) in each analyzed condition (**S2E Fig**), with no prominent differences between ModT(L) and ModT(S) within the common region (**Fig 2D**). This is particularly apparent when subtracting their normalized reactivities (**Fig 2E**). Hence, the DMS probing confirmed the ILP data, indicating that ModT(L) and ModT(S) adopt the same core structures in vitro and in vivo. Generally, we noticed a strong positive correlation across all analyzed samples (≥0.86), which indicates overall similar RNA structures for ModT in different growth conditions (**S2E Fig**). Nevertheless, hierarchical clustering using the single linkage algorithm showed that the samples from the late time points display the lowest reactivity across several regions, while those from the exponential phase (EP) have an overall increased reactivity (**Fig 2D**). Interestingly, higher overall reactivities were also observed for the Proteinase K digested lysates and for the in vitro transcribed RNA samples (**Fig 2D**). Arguably, the key difference between the native in vivo conditions, and the in vitro/deproteinated conditions, respectively, is the absence of proteins in the latter two, which indicates the interaction of ModT with proteins in vivo (**Figs 2D and S2E**).

Specifically comparing reactivity changes of single residues between conditions (e.g., in vivo deproteinated versus in vivo native, (**Figs 2E** and **S2F**) identified strong changes in base-paired regions P7 and P8, and in the regions with the lowest structural confidence across the 3 ModT structure models (P3-P5) (**Fig 2A–2C**). Therefore, we wanted to explore whether our experimental DMS-MaP data of ModT could identify a model with best fit to the ModT structural data. For that, we calculated the ROC-AUC score (receiver operator characteristic–area under the curve), a summary statistics, which serves as a measure of the agreement between the predicted base pairing in a model and the experimentally determined DMS reactivity [31]. Indeed, this analysis clearly favored the updated consensus secondary structure model (**Fig 2A**), as it displayed the highest ROC-AUC score across all tested conditions (**S2G Fig**).

In summary, our data suggest overall identical structures for both isoforms in vivo and in vitro. Furthermore, the identification of a best-fitting structure model provides a crucial step in delineating the structural constraints for ModT function as it will guide the design of mutations in critical base-paired regions in future experiments.

### ModT acts in trans and regulates transition into the stationary phase of growth

**Fig 3:**
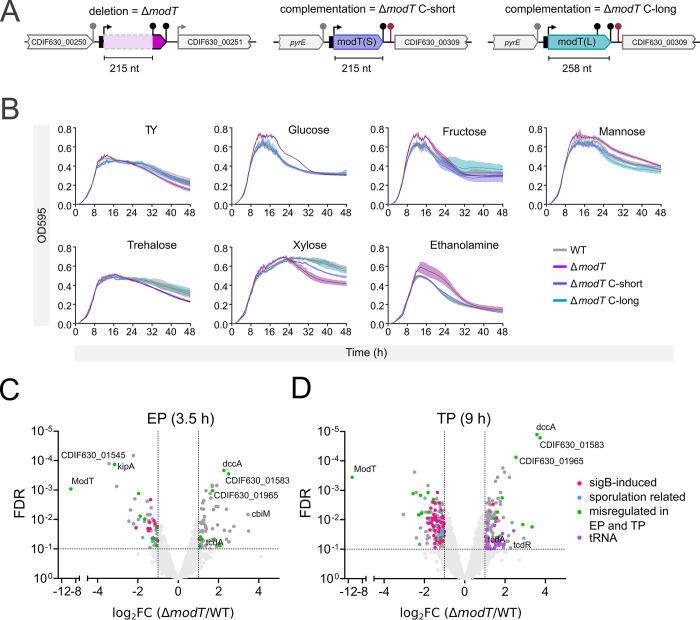
Growth analysis and transcriptomic profiling of *modT* mutants reveal a *trans*-acting function important for the transition into the stationary phase. **(A)** Schematic of chromosomal *modT* deletion, and in trans complementations at the *pyrE* locus of the short and the long isoforms, respectively. **(B)** Growth curves of *C*. *difficile* 630 WT, Δ*modT* and complemented strains in TY medium supplemented with indicated carbon sources. The underlying data can be found in S5 Dataset. **(C, D)** Volcano plots showing transcriptomic comparison between *C*. *difficile* 630 WT and Δ*modT* grown in TYG to EP (3.5 h) and TP (9 h). FDR cutoff was set to <0.1. Genes commonly up- or down-regulated at both time points are highlighted in green. Genes belonging to the SigB regulon [35] are indicated in red, genes induced during sporulation in a Spo0A-dependent manner [39] are labeled blue. Up-regulated tRNA genes are colored in purple. The underlying data can be found in S2 and S5 Datasets. EP, exponential phase; FDR, false discovery rate; TP, transition phase; WT, wild type.

As a first step towards identifying cellular functions of ModT, we deleted the first 215 nt of *modT* (Δ*modT*) corresponding to the conserved ModT(S) isoform (**Fig 3A**). This abolished production of both isoforms without causing any polar effects on neighboring genes (CDIF630_00250 and CDIF630_00251) (**S3A and S3B Fig**). Due to the nutrient-dependent expression profile of ModT, we initially compared growth of wild-type (WT) and Δ*modT* in TY medium supplemented with carbon and energy sources that can be utilized by *C*. *difficile* during gut colonization (**Fig 3B**) [32–34]. The deletion of *modT* was associated with an increased maximum optical density (OD) (**S3C Fig**), which appeared to be the result of extended exponential growth (**Fig 3B**). This overgrowth phenotype was particularly pronounced in media containing hexose monosaccharides (glucose, fructose, and mannose) or ethanolamine. Curiously, the overgrowth did not correlate with the carbon source-dependent ModT expression in late stationary phase, which does not occur during growth on ethanolamine (**Fig 1C**). Nevertheless, it indicates that the presence of ModT in exponential phase of growth is needed for a timely transition into the stationary phase. Importantly, in trans chromosomal complementation of Δ*modT* with either the short or the long isoform resulted in an expression level and pattern similar to wild-type ModT (**S3B Fig**) and restored growth to wild-type level for both complementation strains (**Fig 3B**). This demonstrates that the short isoform is sufficient for ModT function. Furthermore, and in agreement with a recent study in *C*. *acetobutylicum* [22], this experiment rules out a *cis*-acting riboswitch function of ModT, which was originally proposed based on its genetic location and complex secondary structure [15].

To identify cellular pathways that are regulated by ModT, we performed comparative transcriptome analysis. Wild-type and Δ*modT* strains were grown in TY medium supplemented with glucose (TYG). We collected samples in the EP (3, 5 h) and the TP (9 h), when the knockout strain begins to outgrow the wild type (**S3D Fig**). This analysis identified 145 differentially regulated genes in the exponential phase and 344 differentially regulated genes in the TP (each with a false discovery rate (FDR) ≤ 0.1 and a log_2_ FC ≥ 1 or ≤ -1) (**Figs 3C, 3D, and S3E and S2 Dataset**). The list of regulated genes was dominated at both timepoints by metabolic genes and ABC-type sugar and amino acid transporters, which might be linked to the observed carbon-source dependence of ModT expression and the growth phenotype of the mutant.

Given the outgrowth phenotype of Δ*modT*, we focused on differentially expressed genes in the TP. We noticed that deletion of *modT* was associated with broad down-regulation of the σ^B^-dependent regulon. Sigma B regulates the general stress response in *C*. *difficile*, mediating tolerance towards many stress conditions, but also adaptive responses during intestinal colonization [35,36]. In our dataset, approximately 25% (62/248) of the genes that are under positive control of σ^B^ [35], were significantly down-regulated in Δ*modT* during the TP (**S3F Fig and S3 Dataset**). In accordance with a low σ^B^ activity during early exponential phase [36], only 7% of the regulon (16 genes) was down-regulated at this time point.

We further observed down-regulation of genes under the control of σ^H^, a central regulator of the TP [37]. Its regulon is less defined than the σ^B^ regulon, but of the 40 genes known to harbor a σ^H^ promoter [37], 18 (45%) were down-regulated in Δ*modT* during the TP (**S3 Dataset**). Furthermore, we found globally increased tRNA abundance in the mutant, with 75 out of 90 tRNA loci being up-regulated. Given the technical challenges associated with tRNA isolation and sequencing [38], we sought to validate this observation by northern blotting. To that end, we generated a high-resolution time curve covering all growth stages of *C*. *difficile* and probed for 2 different tRNAs (**S3G Fig**). This validated the increased abundance of these tRNAs in the knockout during the TP, in comparison to the wild type. Interestingly, the difference increases at late stationary phase time points due to a reduction of the tRNA fraction in the wild type, while levels are stably maintained in the knockout. Intriguingly, probing for 5S indicated that this trend also occurs with ribosomal RNA.

**Fig 4:**
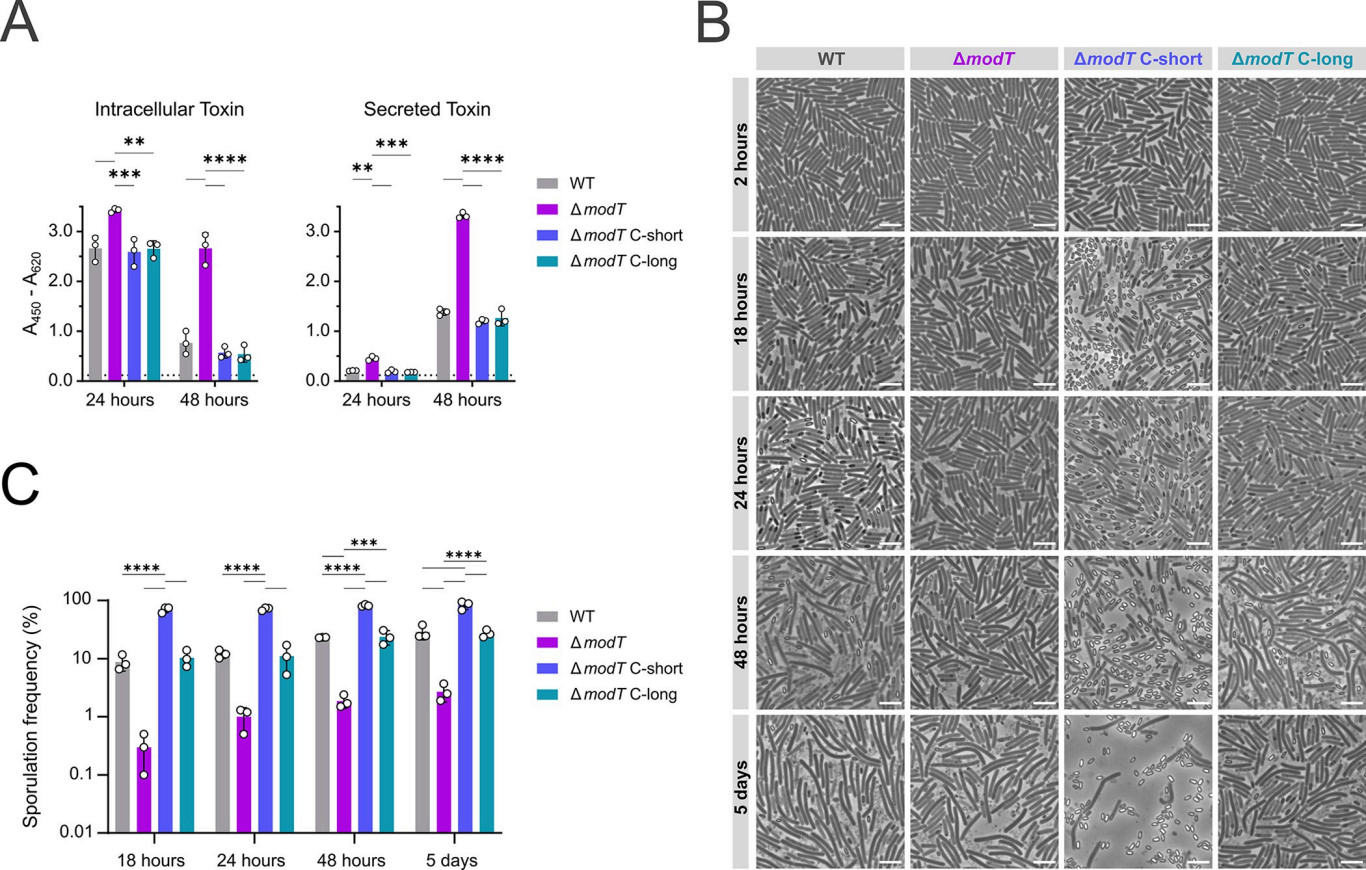
ModT regulates stationary phase associated spore formation and toxin production. **(A)** ELISA-based toxin quantification (TcdA and TcdB combined) in cell lysates (“intracellular toxin”) and cell free supernatant (“secreted toxin”) of *C*. *difficile* 630 WT, Δ*modT* and complemented strains grown in TYG medium. Relative toxin amount is displayed as absorbance at 450 nm (A_450_) normalized by the absorbance at 620 nm (A_620_). *n* = 3 biological replicates. The underlying data can be found in S5 Dataset. **(B)** Representative phase-contrast micrographs (*n* = 3 biological replicates) of *C*. *difficile* 630 WT, and *modT* mutants in 70:30 medium. Time points are indicated relative to the onset of the TP. Scale bar = 5 μm. **(C)** Sporulation frequency calculated from the phase-contrast microscopy displayed in (B). *n* = 3 biological replicates. Time points are indicated relative to the onset of the TP. The underlying data can be found in S5 Dataset. Significance for data shown in (A) and (C) was determined by two-way ANOVA with Tukey’s multiple comparisons test. Only significant differences with *P* < 0.05 are displayed [**P < 0.01, ***P < 0.001, ****P < 0.0001]. TP, transition phase; WT, wild type.

In addition to a broad down-regulation of the σ^B^ and σ^H^ regulons, we identified pathways that directly regulate toxin production and sporulation. Specifically, we observed up-regulation of the *tcdA* gene, encoding for the large clostridial toxin A (log_2_FC = 1.1 and 0.9 in exponential and transition phase, respectively), and of the *tcdR* gene (log_2_FC = 2.3 in TP), encoding for the alternative sigma factor TcdR that positively controls synthesis of the toxin genes *tcdA* and *tcdB*. This indicated that ModT activity may limit toxin production. To test whether the observed transcriptome changes translate into higher toxin levels, we compared these between wild type and mutant. This revealed that the *modT* mutant produced more toxin than the wild type (**Fig 4A**, left panel), which ultimately resulted in higher levels of toxin released into the culture supernatant (**Fig 4A**, right panel). Importantly, as seen for the growth phenotype, complementation of either the short or the long isoform of ModT restored toxin production to wild-type levels.

Furthermore, 23 sporulation genes [39] were down-regulated in TP (**Fig 3D and S3 Dataset**), including those encoding for the sporulation sigma factors σ^E^ (log_2_FC = −1.1), σ^F^ (log_2_FC = −0.92), and σ^G^ (log_2_FC = −1.05), all of which coordinate spore developmental steps downstream of the master regulator of sporulation Spo0A. Therefore, we quantified sporulation frequencies in wild-type, Δ*modT* and complemented strains over an extended period of 5 days. Spore enumeration by phase contrast microscopy revealed a significant decrease in sporulation frequency in the knockout (**Fig 4B and 4C**), which was independently validated by CFU-based calculation of vegetative cells and ethanol-resistant spores (**S4A and S4B Fig**). This sporulation defect remained throughout the experiment, indicating that sporulation is not just delayed in Δ*modT*, but permanently repressed. Surprisingly, while chromosomal complementation of Δ*modT* with the long isoform (Δ*modT* C-long) reconstituted sporulation to wild-type levels, complementation with the short isoform (Δ*modT* C-short) resulted in a pronounced hyper-sporulation phenotype (**Figs 4B, 4C, S4A, and S4B**). Northern blot validation of ModT revealed both a mild increase in ModT(S) transcript levels and a prolonged expression of ModT(S) at later time points in the short complementation strain, relative to the WT or Δ*modT* C-long (**S4C Fig**). This either suggests that small alterations in ModT abundance and/or presence of ModT in stationary phase can have a strong impact on sporulation, or that the 2 isoforms differ in their capacity to regulate sporulation.

Taken together, the extended exponential growth and the global down-regulation of the σ^B^ and σ^H^ regulons in Δ*modT* indicates that ModT regulates the transition from exponential to stationary phase of growth in *C*. *difficile*. Concomitantly, we observe changes in spore formation and toxin production, 2 virulence-determining processes that are tightly regulated in response to the physiological state of the cell.

### ModT regulates sporulation by modulating cellular cyclic di-GMP levels

**Fig 5:**
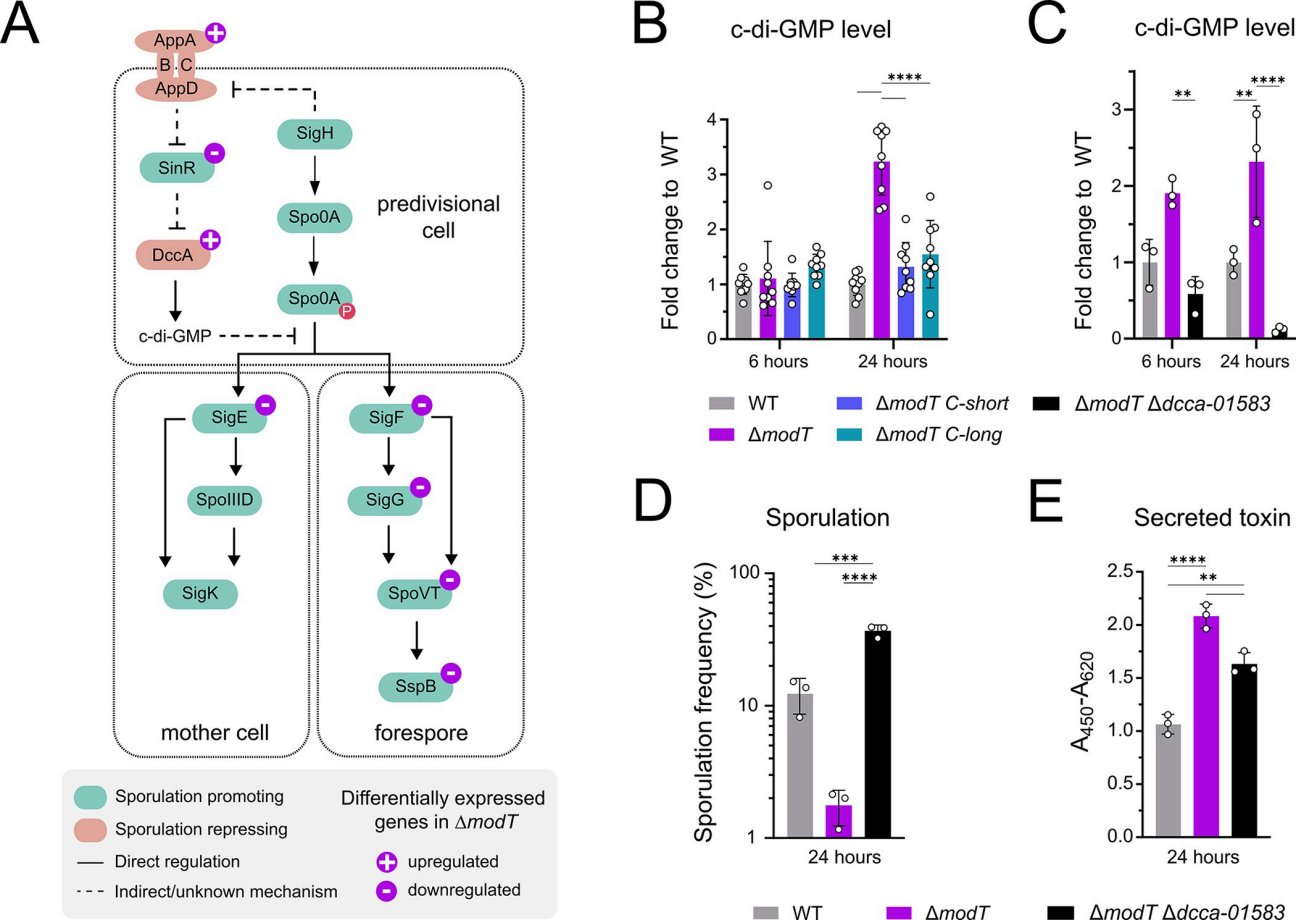
ModT modulates sporulation and toxin production via changes in cellular c-di-GMP levels. (A) Schematic overview of differentially expressed genes in Δ*modT* that were shown to be involved in the regulation of spore formation in *C*. *difficile* [40–42,56,65]. (B) Changes in c-di-GMP levels in Δ*modT* and in complemented strains grown in TYG medium. Measurements were done using a fluorescent c-di-GMP type II riboswitch reporter. Values in the Δ*modT* and in complemented strains were calculated as fold-change relative to the *C*. *difficile* 630 WT signal. Data represent the mean of 3 independent experiments performed with *n* = 3 biological replicates per experiment (total *n* = 9). The underlying data can be found in S5 Dataset. (C) c-di-GMP levels in WT, Δ*modT* and Δ*modT*Δ*dccA-01583*. Measurements were done as in (B) Values in the Δ*modT* and Δ*modT*Δ*dccA-01583* strains were calculated as fold-change relative to the *C*. *difficile* 630 WT signal. The underlying data can be found in S5 Dataset. (D) Assessment of sporulation frequency in 70:30 medium by phase-contrast microscopy. The underlying data can be found in S5 Dataset. (E) Relative quantification of toxin (TcdA and TcdB combined) as determined by ELISA in cell free supernatants of *C*. *difficile* 630 WT, Δ*modT* and Δ*modT*Δ*dccA-01583* grown in TYG medium. Each experiment was performed in 3 (*n* = 3) biological replicates. The underlying data can be found in S5 Dataset. For the experiments shown in (B) and (C), the fluorescence values were normalized to the control (pRS^A>G^) as described in S5B Fig. For data shown in (B) and (C), significance was determined by two-way ANOVA with Tukey’s multiple comparisons test; for data shown in (D) and (E), one way ANOVA with Tukey’s multiple comparisons test was applied. Only significant differences with *P* < 0.05 are displayed [**P < 0.01, ***P < 0.001, ****P < 0.0001]. WT, wild type.

We sought to better understand the mechanistic basis for the observed changes in toxicity and sporulation in the *modT* mutant. Our transcriptome analysis highlighted several differentially expressed genes shown to affect sporulation (highlighted in **Fig 5A**). Among them, the *dccA* gene caught our attention, because it was the top up-regulated transcript at both time points in Δ*modT* (**Fig 3C and 3D and S2 Dataset**). DccA is a diguanylate kinase containing a GGDEF domain that catalyzes the synthesis of the second messenger c-di-GMP [40]. Overexpression of DccA was shown to suppress early sporulation events, downstream of Spo0A, by increasing cellular c-di-GMP levels [41] through a yet unknown mechanism. Increased *dccA* transcription, and consequently, increased c-di-GMP levels, have been associated with a sinRR’ deletion mutant which is asporogenous and displays global gene expression changes in pathways regulating sporulation [42]. Aligning with that, the bicistronic operon encoding SinR and its protein antagonist SinR’ [42], was down-regulated in Δ*modT* at both time points (**Fig 5A**). Hence, our transcriptome analysis of the *modT* mutant (**S2 Dataset**) suggested that the pronounced effects on sporulation could be linked to changes in cellular levels of c-di-GMP.

To investigate this link, we assessed whether deletion of ModT was associated with increased cellular c-di-GMP levels. For this, we adopted a reporter system in which a c-di-GMP sensitive riboswitch represses transcription of a downstream encoded mCherry protein at low c-di-GMP levels [43–45] (**S5A Fig**). Hence, increasing mCherry fluorescence serves as a proxy for increased c-di-GMP levels. To control for background fluorescence, we employed a reporter with an A70G mutation (pRS^A70G^) that renders the riboswitch unresponsive to c-di-GMP. Wild-type, Δ*modT* and the complemented strains, each harboring the different reporter constructs and an empty vector control, were grown in TYG medium to mid-exponential (6 h) and stationary phase (24 h), and mean fluorescence intensities were assessed by flow cytometry (**S5B Fig**). Comparing the mean fluorescence in each strain background carrying the functional reporter (pRS), the mutated reporter (pRS^A70G^), and the empty vector control (pEV), revealed some residual fluorescence activity for the mutated reporter (**S5B Fig**). Therefore, all fluorescence data were normalized to the background fluorescence of the same strain carrying the mutated reporter construct (**Fig 5B**). There were no overall significant differences between the WT and the Δ*modT* strain in the early stages of growth. However, at 24 h, we consistently detected increased fluorescence in the *modT* mutant. Importantly, this increase in reporter activity could be reverted to WT levels in both complemented ModT strains (**Fig 5B**). This confirmed our initial hypothesis that deletion of *modT* leads to increased cellular c-di-GMP levels, presumably through increased levels of DccA.

The *dccA* gene is co-transcribed with CDIF630_01583, which is a putative diguanylate kinase signaling protein. However, it also contains several potential sensory domains of the Per-Arnt-Sim (PAS) type, in addition to an EAL-domain which may degrade c-di-GMP [46]. The regulatory impact of CDIF630_01583 has not been studied so far. Since the entire operon was up-regulated in our dataset, we wanted to test if and how CDIF630_01583-*dccA* up-regulation was contributing to the changes in sporulation in the *modT* mutant. For that, we deleted the *dccA-01583* operon in Δ*modT* and compared the fluorescence of the resulting double knockout to Δ*modT* and the wild type. This revealed that c-di-GMP levels did not increase in a Δ*modT* Δ*dccA-01583* double knockout, and even dropped below WT levels (**Fig 5C**). Consequently, deletion of the *dccA-01583* operon in the Δ*modT* strain background did not just restore sporulation frequency to WT levels, but actually increased the frequency (**Fig 5D**). Taken together, these data show that *modT* deletion leads to sporulation inhibition via increased DccA-mediated c-di-GMP synthesis. In contrast to sporulation, we observed only partial restoration of toxin production in the Δ*modT* Δ*dccA-01583* double knockout (**Fig 5E**), suggesting additional, c-di-GMP-independent, regulatory mechanisms contributing to the observed increases in toxin production in Δ*modT*.

In summary, the effects of ModT on spore formation are mediated through the ubiquitous second messenger c-di-GMP.

### ModT orthologs from distant phylogenetic families are functionally conserved

**Fig 6:**
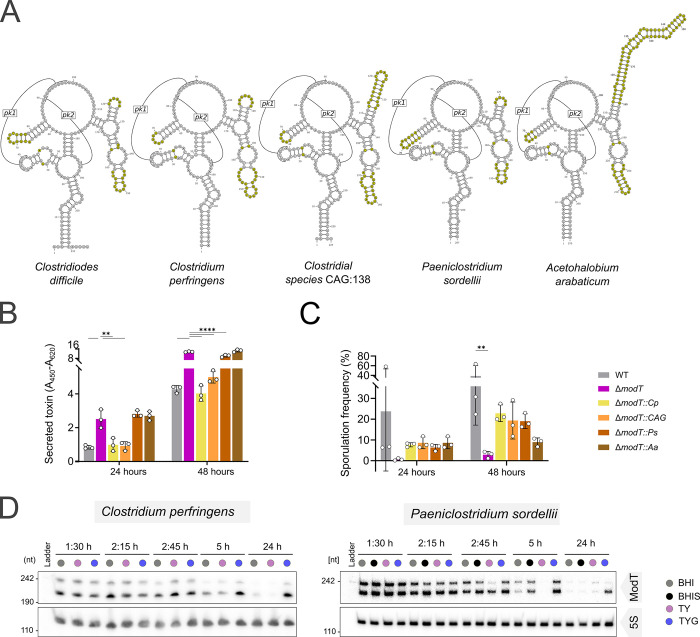
Orthologs from distant phylogenetic families can replace ModT function in *C*. *difficile*. **(A)** Consensus secondary structure models of ModT orthologs used for complementation. Regions varying in primary sequence and secondary structure across all orthologs are highlighted in yellow. **(B)** Relative quantification of toxin (TcdA and TcdB combined) determined by ELISA in cell free supernatant of WT, Δ*modT* and *C*. *difficile* with ModT ortholog replacements grown in TYG medium. Each experiment was performed in 3 (*n* = 3) biological replicates. The underlying data can be found in S5 Dataset. **(C)** Assessment of sporulation frequency in 70:30 medium based on phase-contrast microscopy images (*n* = 3 biological replicates) shown in **S6D Fig**. The underlying data can be found in S5 Dataset. **(D)** Northern blot-based expression analysis of ModT in *C*. *perfringens* and *P*. *sordellii* across different growth stages in various rich media, including BHI, BHI + yeast extract (BHIS), Tryptone Yeast (TY), and TY supplemented with 0.5% glucose (TYG). 5S rRNA served as loading control. Depicted is a representative of 3 biological replicates. Significance for data shown in (B) and (C) was determined using two-way ANOVA with Dunnett’s multiple comparisons test. Only significant differences with *P* < 0.05 are displayed [**P < 0.01, ***P < 0.001, ****P < 0.0001]. BHI, brain heart infusion; WT, wild type.

Given the high sequence conservation of ModT family members, we wanted to explore whether ModT orthologs from diverse species are capable of complementing ModT function in *C*. *difficile*. To this end, we exchanged the sequence of the consensus ModT motif (first 215 nt) in *C*. *difficile* (**S6A and S6B Fig**) with the consensus motif of 3 ModT orthologs from the phylum Bacillota, including *Clostridium perfringens* and unclassified *Clostridium* sp. CAG:138 (genus *Clostridium*, family *Clostridiaceae*), as well as *Paeniclostridium sordellii* (genus *Paeniclostridium*, family Clostridiaceae). In addition, we chose 1 ortholog from the phylum Actinomycetota, *Acetohalobium arabaticum* (genus *Acetohalobium*, family Halobacteroidaceae). All 4 orthologs possess core structural features of the ModT family, but vary in size and sequence, particularly in the regions of the variable P5, P7, and P8 stems (**Figs 6A and S6A**). For example, while the P7 and P8 stem regions comprise only ~51 nt in *C*. *difficile*, they are 73 nt and 118 nt long in CAG:138 and *A*. *arabaticum*, respectively. Furthermore, the primary sequences of the P5 loop vary in all strains and contain covarying mutations in various places. Northern blot validations of ModT transcripts in complemented strains showed that the orthologs from *C*. *perfringens* and CAG:138 are expressed at levels similar to WT ModT, although the ratios between short and long isoform vary (**S6C Fig**). In contrast, strains expressing orthologs from *P*. *sordellii* and *A*. *arabaticum* showed low ModT levels (**S6C Fig**). Despite expression level differences, we assessed toxin production and sporulation frequency in all complemented strains to determine whether these orthologs can replace ModT function in *C*. *difficile*. In comparison to the Δ*modT* mutant, the orthologs of *C*. *perfringens* and CAG:138 were able to fully restore both toxin production and sporulation frequency to WT levels (**Figs 6B, 6C, and S6D–S6F**). *P*. *sordellii* ModT was able to restore sporulation frequency, whereas toxin levels remained comparable to Δ*modT*. Similarly, complementation with ModT from *A*. *arabaticum* was able to partially rescue sporulation but did not restore toxin production. The fact that *P*. *sordellii* and *A*. *arabaticum* orthologs can only partially rescue the Δ*modT* associated phenotypes might simply be due to their weaker expression in C. *difficile*, but it could also be an indication for a functional divergence.

To develop a better understanding of whether ModT orthologs carry out conserved functions, we sought to explore their expression and isoform profiles within their native hosts. For that purpose, we chose the 2 human pathogens *Clostridium perfringens* and *Paeniclostridium sordellii* that belong to the family Clostridiaceae. Although they are phylogenetically distant from *C*. *difficile* (family Peptostreptococcaceae), they are both spore formers and encode homologs of the large clostridial toxins TcdA and TcdB [47,48]. Both bacteria were grown in media with varying levels of glucose to collect time-resolved expression profiles across the different growth stages. This expression analysis revealed several interesting parallels to *C*. *difficile* ModT (**Fig 6D,** compare to **Fig 1C**). Firstly, both orthologs occur in 2 isoforms, one matching the conserved ModT motif (~210 nt) and a larger isoform of ~240 nt. Secondly, *modT* is also strongly expressed during exponential growth, but depleted in the stationary phase. Third, medium supplementation with glucose extends the expression of the RNAs into the stationary phase. Although a recent expression analysis of ModT in *C*. *acetobutylicum* did not analyze the effect of different carbon sources on ModT expression, it also showed that ModT transcript levels decrease after entry into stationary phase [22]. In addition, the promoter regions of *modT* in all 4 clostridial species contain a highly conserved sigA consensus motif (**S6G Fig**), strongly suggesting that SigA-mediated *modT* expression in the exponential phase is widespread and may be a key aspect of its regulation. Despite these similarities, there is one major difference in that ModT(S) isoforms appear more stable than the ModT(L) isoforms in *C*. *perfringens* and *P*. *sordellii*, while the long isoform is lacking entirely in *C*. *acetobutylicum*.

In summary, the conserved expression profiles in various bacteria, along with the capacity of orthologs to complement ModT activity in *C*. *difficile* suggests that ModT members from diverse phylogenetic backgrounds are functionally conserved.

## Discussion

Broadly conserved, structured noncoding RNAs are central regulators of fundamental cellular processes in bacteria. Our characterization of the ModT RNA in *C*. *difficile* echoes this notion as its deletion results in pronounced phenotypes under standard growth conditions. These comprise delayed transition from the exponential to the stationary phase of growth, reduced spore formation, and increased toxin production. Our results align with a recent study, which showed that deletion of *modT* results in sporulation and aggregation deficiency in *C*. *acetobutylicum* [22]. These findings strongly suggest a conserved role for ModT in regulating lifestyle transitions in different bacterial species.

Here, we show that ModT regulates this lifestyle transition, at least in part, through modulating cellular levels of the second messenger molecule c-di-GMP. We show that deletion of *modT* is associated with increased cellular levels of c-di-GMP, and we were able to causally link this increase in c-di-GMP to the observed sporulation defect in the *modT* mutant. Our results complement previous findings that the overexpression of *dccA*, and associated increases in cellular c-di-GMP, lead to significant decreases in sporulation frequencies [41]. These findings are intriguing because c-di-GMP is a nearly ubiquitous bacterial second messenger that regulates fundamental processes such as bacterial growth and behavior (motility, aggregation, and biofilm formation), but also developmental processes, such as spore formation, in diverse bacteria [49].

In fact, regulation of c-di-GMP levels by ModT might explain the sporulation and aggregation defects observed in a *modT* mutant in *C*. *acetobutylicum* [22], as both are known c-di-GMP-dependent processes in bacteria [49]. In the future, it will be interesting to see if ModT orthologs generally impact biological processes that are regulated by c-di-GMP.

Toxin production in *C*. *difficile* is also a c-di-GMP-dependent process, but the current model for c-di-GMP mediated regulation of toxin production [50,51] does not align with the increased toxin production in the *modT* mutant. That is, c-di-GMP was shown to negatively regulate toxin production by mediating the riboswitch-dependent premature transcription termination of the flagella operon at high c-di-GMP levels. As a consequence, sigD, which drives TcdR production and which is encoded in the flagella operon, is repressed. In our transcriptome analysis, we do not observe such down-regulation of the flagellar operon, which suggests that the toxin phenotype is driven by c-di-GMP-independent changes in the *modT* mutant. In line with this, we observed only partial restoration of toxin production in the Δ*modT* Δ*dccA-01583* double knockout. However, we would like to point out that sporulation assays and toxin quantification were performed in different media. Thus, to dissect the relative contribution of ModT-mediated changes in c-di-GMP levels to toxin production and spore formation, and to understand how both processes effect each other in the modT mutant, further experiments are required, e.g., under conditions when both sporulation and toxin production occurs.

Curiously, the identified stationary phase phenotypes of *modT* mutant strains (sporulation, aggregation) seem to be inversely correlated with the largely conserved expression of ModT during the exponential phase of growth in several clostridial species. In fact, *C*. *difficile* ModT is among the most abundant and most stable RNAs in the cell during vegetative growth, both in *C*. *difficile* and *C*. *acetobutylicum*. However, we show that its levels drop almost completely upon transition of bacteria into the stationary phase in *C*. *difficile*, *C*. *perfringens*, and *P*. *sordellii*. This suggests that the presence of ModT might be needed to initiate the timely transition from exponential to stationary phase of growth. In this context, the ModT mediated phenotypes could be interpreted as a failure to sense and/or transmit a stress signal, resulting in the observed prolonged vegetative growth of the mutant. The sporulation defect might subsequently result from a lack of energy to produce spores. Likewise, the up-regulation of toxin production could indicate an inadequate coordination between central metabolic pathways and the regulation of toxin genes in the *modT* mutant.

An interesting feature of ModT that stood out to us is the existence of a longer isoform that differs from the short isoform through a 3′ extension. Due to a lack of primary sequence conservation in the extended 3′-end, it was missed in the original genome-based annotation by Weinberg and colleagues [15]. Based on our data, we propose that ModT(L) is the result of read-through transcription into an intrinsic terminator downstream of ModT(S) in *C*. *difficile*. This longer isoform does not appear to be specific to *C*. *difficile*, because we detected it in *C*. *perfringens* and *P*. *sordellii* as well. That said, a recent expression analysis of the ModT ortholog in *C*. *acetobutylicum* [22] did not reveal the existence of a long isoform, which does raise the question how common it might be. To gather some clues, we revisited the original conservation analysis [15] which contained in silico terminator predictions for orthologous ModT sequences, including *C*. *difficile*. These analyses predict rho-independent transcription terminators in close proximity to the 3′-end of ModT(S) for all 3 clostridial species expressing a long ModT isoform but not for ModT from *C*. *acetobutylicum* ([15], supplemental files). Rho-independent transcriptional terminators are complex structures that comprise both a stem-loop and a poly-uridine stretch which suggests that they do not evolve easily. Therefore, it seems more plausible that intrinsic terminators were originally present in all ModT orthologs, but have been lost over time in individual bacteria.

Are the 2 isoforms just the result of transcriptional read-through events or do they confer distinct functions in those organisms where they are produced? Although this needs to be carefully investigated in the future, our analyses in *C*. *difficile* provide initial evidence for potential functional divergence between both isoforms. That is, although our structure probing data suggest that both isoforms adopt a highly similar structure, the in vivo half-lives differ markedly between ModT(L) and ModT(S). The increased stability of ModT(L) suggests that the stable secondary structure of the terminator mediates protection against 3′–5′ exonuclease activity [52]. However, it could also indicate engagements of both isoforms in different ribonucleoprotein complexes. Furthermore, complementation with the short, but not with the long isoform, leads to a striking hyper-sporulation phenotype, even though both isoforms restore c-di-GMP to a level comparable to the WT. This suggests that ModT(S) might carry out additional roles that impact sporulation frequency in a c-di-GMP independent manner, possibly through interactions with other protein regulators. This hypothesis could be tested in the future, by performing isoform-specific pulldowns of ModT to identify interacting protein and RNA partners.

But what is the underlying molecular function of ModT? The broad sequence conservation across the entire ModT RNA implies a function that is not linked to the simple base-pairing mechanism of small regulatory RNAs. Their primary sequence conservation is typically very narrow, and mostly order or genus specific [8]. Sequence conservation is usually restricted to the seed region of complementarity to their regulated target mRNAs. Furthermore, our previous analyses of the two known sRNA-binding proteins in *C*. *difficile*, Hfq and KhpB, did not reveal binding of ModT to either of these 2 global RBPs [21,26,53]. Therefore, based on our current knowledge, it seems unlikely that ModT acts as a canonical sRNA.

Broadly conserved bacterial ncRNAs with intricate secondary and tertiary structure, such as ModT, often act as riboswitches or ribozymes. Our study confirms recent findings in *C*. *acetobutylicum* that phenotypes of *modT* knockout strains can be complemented in *trans* through expression of *modT* from a plasmid or an independent genomic locus, thereby ruling out a *cis*-acting riboswitch function for ModT [22]. These data, however, do not exclude the possibility that ModT has evolved from an ancient riboswitch to carry out *trans*-regulatory functions. Curiously, our in vivo DMS-MaP data show changes in reactivities in ModT between the exponential and the transition/stationary growth phases which opens the possibility that ModT still acts like a “riboswitch” in that it binds a ligand which induces conformational changes in ModT. Binding assays with potential ligands, such as c-di-GMP, combined with DMS-MaP analyses should provide more insights into this hypothesis in the future.

Other conserved RNA classes with intricate tertiary structures are catalytic RNAs, called ribozymes [5]. Almost all described ribozymes are self-cleaving RNAs, the only exception is RNase P which acts on other RNA substrates, and is involved in pre-tRNA processing [9]. However, in light of the increased levels of tRNAs and 5S RNA in the *modT* knockout strain, it is an intriguing, yet speculative, thought that ModT might be involved in the processing of other RNAs, including tRNAs and rRNAs. Therefore, future experimental efforts should focus on both potential self-cleaving and *trans*-cleaving activities of ModT. Some ribozymes, such as the glmS ribozyme, catalyze RNA cleavage in response to binding a specific metabolite [54]. Therefore, these cleaving assays should be performed in the presence of potential ligands such as c-di-GMP.

Interestingly, in about approximately 20% of all ModT species (including *C*. *difficile*), the P8 stem displays an E-loop structure [15,22], that also occurs in ribosomal RNAs as recognition sites for RNA–protein interactions. This may indicate that at least some ModT orthologs engage in protein interactions via this structural element [55]. Support for this hypothesis comes from our previous Grad-seq (gradient profiling by sequencing) analysis in *C*. *difficile* 630, to predict RNA and protein complexes based on glycerol gradient sedimentation profiles [26]. This revealed sedimentation of both ModT isoforms in molecular weight fractions that indicate their association with a protein (or protein complex). Furthermore, our DMS-structure probing analysis indicated increased reactivities in several regions of the RNA in deproteinated versus native cell lysates. Our current selection of ModT orthologs for functional complementation of the *modT* knockout in *C*. *difficile* did not contain an ortholog lacking the E-loop motif. Hence, in the future it will be interesting to determine whether orthologs lacking this motif can equally complement all phenotypes in *C*. *difficile*, and whether this motif contributes to RNA-protein interactions.

In summary, our findings establish ModT as a conserved regulator of the bacterial life cycle and provide experimental avenues towards delineating the molecular mechanism of its intricate RNA structure.

## Materials and methods

### Bacterial strains and growth conditions

A list of bacterial strains used in this study is provided in **S4 Dataset**. *C*. *difficile* strain 630 (DSM 27543) cultures were routinely grown at 37°C inside a Coy chamber (85% N_2_, 10% H_2_, and 5% CO_2_) on brain heart infusion (BHI) agar plates (1.5% agar) or in autoclaved BHI broth (37 g/l). Petri dishes were anaerobically incubated for at least 4 h prior to inoculation, while liquids were kept in the Coy chamber overnight to allow diffusion of entrapped oxygen. For selection of plasmid-harboring *C*. *difficile* 630, antibiotics were added at the following concentrations: thiamphenicol 15 μg/ml, cefoxitin 8 μg/ml, cycloserine 250 μg/ml. Cryostocks were stored at −80°C in BHI broth containing 15% glycerol.

To cultivate *C*. *difficile* with different carbon and energy sources autoclaved TY medium (30 g/l tryptone (Roth), 20 g/l yeast extract (Roth)) was supplemented with the respective source from 20% (w/V) sterile filtered stock solutions in ddH_2_O to a final concentration of 0.5%. For growth curves, TY day cultures were inoculated to an OD_600_ of 0.05 from TY overnight cultures, grown to exponential phase (OD_600_ ~0.5) and subsequently diluted to an OD_600_ of 0.01 in TY containing the respective carbon source. For growth curves in BHI and BHIS medium (BHI with 5 g/l of yeast extract (Roth)), cultures were inoculated from exponential phase BHI cultures. Optical density at 600 nm was recorded in a transparent 96-well plate in a Biotek H1 microplate reader.

*C*. *perfringens* ATCC 133124 and *P*. *sordellii* ATCC 9714 were cultured under the conditions indicated for *C*. *difficile* 630, with the following changes: For growth of *P*. *sordellii* in BHIS medium, 10% w/V sterile filtered cysteine solution was added to a final 0.1% (w/V) after autoclaving. For *C*. *perfringens*, the overnight culture was replaced with a day culture. *Escherichia coli* (*E*. *coli*) strains were aerobically cultivated in autoclaved Luria–Bertani (LB) broth (10 g/l tryptone, 5 g/l yeast extract, 10 g/l NaCl) at 37°C with shaking at 220 revolutions per minute (RPM), or on LB-agar (1,5% agar). Plasmids were maintained by adding 20 μg/ml chloramphenicol as a selection antibiotic. Cryostocks were stored at −80°C in LB broth with 25% glycerol.

### Toxin ELISA

For *C*. *difficile* toxin quantification, 2.5 OD_600_ units of bacterial culture were centrifuged at 10,000 × g, 4°C for 10 min. The supernatant was transferred to a new tube and centrifuged again under the same conditions to remove any residual cells. Approximately 250 μl cell free supernatant was stored at −80°C until quantification of the “secreted toxin.”

The cell pellet (used for quantification of “intracellular toxin”) was washed with 1 ml PBS (phosphate-buffered saline), then centrifuged again to remove the remaining liquid. Samples were kept at −80°C until further processing.

Cell pellets were suspended in 500 μl of ddH_2_O and transferred to a new tube containing 500 μl 0.1 mm glass beads. For lysis, samples were ground in pre-cooled adapters (−20°C) in a Retsch Mixer Mill at 30 Hertz for 10 min, and subsequently centrifuged at 10,000 × g, 4°C for 15 min to remove debris and beads; 200 μl of the clear supernatant was transferred to a fresh tube and the protein concentration was estimated with a Bradford assay using RotiNano Quant solution (Roth) according to the manufacturer’s instructions. For relative toxin quantification, 4 μg of protein from the “intracellular toxin” sample were used as input, while “secreted toxin” was assessed from 50 μl of the cleared supernatant. The relative amount was determined by toxin ELISA (TGC-E001-1, TGC Biomics) performed and evaluated according to the manufacturer’s instructions. ELISA signal was quantified with a Tecan infinite 200 Pro plate reader assessing absorption at 450 nm and 620 nm. As a final readout, the signal at 620 nm was subtracted from the 450 nm signal.

### Sporulation assays

Sporulation assays were performed in 70:30 sporulation broth medium according to Edwards and colleagues with minor alterations [56]. Overnight TY cultures were used to inoculate day cultures to an OD_600_ of 0.05 in BHIS containing 0.5% fructose and 0.1% taurocholate. After growth to mid-exponential phase, 70:30 medium was inoculated to an OD_600_ of 0.01.

At the end of transition phase (approximately 8 to 10 h after inoculation), the total CFUs from untreated cultures were assessed by serial dilution and spotting in BHIS, and in addition, an aliquot treated with ethanol for 30 min was serially diluted on BHIS agar with 0.1% taurocholate to check for spore carryover. This time point is referred to as 0 hours. At the subsequent time points, vegetative CFUs were assessed by serial dilution and plating on BHIS agar and spores were quantified from culture treated with ethanol (EtOH) (final concentration 48% v/V) for 30 min, followed by serial dilution in PBS and plating on BHIS containing 0.1% taurocholate.

For a second independent analysis at all time points, 500 μl culture aliquots were concentrated by brief centrifugation and approximately 3 μl of the concentrated cells were spotted onto an agarose pad on a glass slide for microscopy. Spores were visualized by phase contrast microscopy using a HC PLAN FLUOTAR 100×/1.32 PH3 oil immersion objective on a LEICA DM2500 microscope. Two fields of view were acquired per biological replicate from which at least 300 cells per field were counted to determine the sporulation frequency. In both methods, sporulation frequency was calculated as [spore count] / [spore count + vegetative cell count] = sporulation frequency.

### Transformations

Plasmids were transformed to *E*. *coli* Top10 by applying a heat shock for 1 min at 42°C. After chilling on ice for 2 min, bacteria were incubated at 37°C, 350 RPM for 1 to 4 h in LB medium without antibiotics and were subsequently plated on selective LB-agar.

For transformations into *E*. *coli* CA434, 80 μl of a bacterial suspension was thawed on ice and mixed with 1 to 2 μl plasmid in a pre-chilled electroporation cuvette. Plasmid was delivered with an electric pulse (1.8 kV, 200 Ω, 4–5 s). Bacteria were recovered at 37°C in LB broth at 350 RPM for 1 h and subsequently streaked on selective LB agar.

### Plasmid conjugation to *C*. *difficile* 630

For conjugations, 1 ml of an *E*. *coli* CA434 overnight culture harboring the respective plasmid was centrifuged at 4,000 × g, RT for 2 min. The pellet was transferred into the Coy chamber, and resuspended in 200 μl overnight culture of the respective *C*. *difficile* strain. After spotting onto BHI agar and 8 h of incubation at 37°C, cells were scraped from the plate with approximately 700 μl BHI broth and restreaked on BHI agar containing antibiotics to select for bacteria harboring the plasmid. Colonies appeared after 24 to 72 h and were restreaked to purity on selective BHI agar plates.

### Plasmid assemblies

All used and generated plasmids are listed in **S4 Dataset.**

#### Deletion of *modT* in *C*. *difficile* 630 (pFF-173)

For allele exchange cassettes, approximately 1.2 kB of the genome regions flanking the 215 nt long knockout site were PCR-amplified from *C*. *difficile* 630 genomic DNA with FFO-923/926 and FFO-924/927. Fragments were merged by SOEing PCR, gel-purified and subsequently digested using SacI (Thermo Scientific) and BamHI (Thermo Scientific). The fragment was ligated into equally digested, gel-purified pJAK112 [21] using T4 DNA ligase (Thermo Scientific), yielding pFF-173.

#### In trans-complementations of *C*. *difficile* 630 *modT* into the neutral *pyrE* locus (pFF-221/222)

For chromosomal complementation into the *pyrE*-locus, *modT* including the native promoter sequence was amplified from *C*. *difficile* 630 gDNA using FFO-1088/1034 (short isoform) or FFO-1089/1034 (long isoform). The vector backbone was amplified from pJAK080 [21] using FFO-1086/1087, which included the sequence of the *slpA* terminator to prevent read-through transcription of *modT* upon chromosomal integration. The vector and inserts were digested using KpnI (Thermo Scientific), gel-purified and subsequently ligated with T4 DNA ligase resulting in the plasmids pFF-221 (short isoform) and pFF-222 (long isoform).

#### Replacement of *C*. *difficile* 630 *modT* with orthologues (pFF-314/320/321/377)

To replace the short *C*. *difficile* 630 *modT* sequence in its native locus, orthologue sequences were PCR-amplified from the respective species’ genomic DNA, using FFO-1294/FFO-1297 for *C*. *perfringens* ATCC13124, FFO-1443/1444 for *P*. *sordellii* ATCC 9714, FFO-1305/1306 for *Acetohalobium arabaticum* DSM 5501 and FFO-1503/1521 for the *Clostridium species* CAG138. Up- and downstream allele exchange cassettes were amplified from *C*. *difficile* 630 genomic DNA using FFO-923/1295 and FFO-924/1296 for *C*. *perfringens*, FFO-923/1445 and 924/1442 for *P*. *sordellii*, FFO-923/1303 and FFO-924/1304 for *A*. *arabaticum*, and FFO-923/1502 and FFO-924/1506 for the up- and downstream allelic exchange cassettes of *C*. *species* CAG138. Respective insert and cassettes were combined to a single fragment by SOEing PCR, digested with SacI and BamHI and ligated into similarly digested, gel purified pJAK112, yielding pFF-320, pFF-314, pFF-321, and pFF-377.

#### In-frame deletion of the CDIF630_01583-*dccA* operon (pFF-451)

For in-frame deletion of the CDIF630_01583-*dccA* operon, homology cassettes were generated using FFO-1733/1741 and FFO-1740/1742. The cassettes were joined by SOEing PCR using FFO-1733/1740, digested with KpnI and SacI to generate sticky ends, and cloned into similarly digested, gel-purified pJAK112.

#### Cyclic di-GMP reporter (pFF-458/441/442)

As a first step to generate a c-di-GMP responsive reporter plasmid, the riboswitch region from the *pilA1* 5′-UTR was amplified from *C*. *difficile* 630 genomic DNA using FFO-1706/1707 introducing an XbaI restriction site to the 5′-end and an SacI restriction site at the 3′-end. The fragment and pFF-380, a plasmid with a pDSW1728 backbone, containing an fdxA-promoter followed by an XbaI restriction site, located upstream of mCherry and a SacI restriction site downstream of the mCherry start codon, were digested with SacI and XbaI and subsequently ligated, creating a reporter system lacking a start codon for mCherry, pFF-458 (no fluorescence control, pEV).

In a second step, mCherry was PCR-amplified from the same construct with FFO-1710/861, appending a Shine–Dalgarno sequence and a start codon to mCherry and moving the SacI restriction site upstream of the translation start site. pFF-458 and the PCR fragment were digested with SacI and BamHI, gel purified and subsequently ligated, generating pFF-441 (c-di-GMP sensitive reporter, pRS).

To generate an inactive riboswitch control, the *pilA1* 5′ UTR was amplified from *C*. *difficile* 630 gDNA in 2 fragments using FFO-1706/1708 and FFO-1709/1707 introducing an A to G mutation that makes the Riboswitch insensitive to c-di-GMP [44,45]. The fragments were unified by SOEing PCR and both fragment and parent vector pFF-441 were digested with SacI and XbaI (Thermo Scientific) and subsequently ligated resulting in pFF-442 (inactivated c-di-GMP reporter, pRS^A70G^).

### Chromosomal manipulation of *C*. *difficile* 630

For chromosomal manipulation, the two-step allelic exchange method was applied as described previously [21,57].

After plasmid conjugation, *C*. *difficile* 630 was restreaked at least twice and subsequently screened for single crossover events. Integrations into the *modT* locus (pFF-173/314/320/321/377/378) were identified by PCR using FFO-360/498, FFO-361/499 and FFO-498/499 and FFO-360/613. Integrations into the *pyrE* locus (pFF-221/222) were identified using FFO-361/612 and FFO-612/613. Single crossover events for generating Δ01583-*dccA* (pFF-451) were identified using FFO-1746/361 and FFO-1743/360.

To allow the second recombination event, single crossover integrants were incubated on BHI-agar for approximately 72 h, harvested in 900 μl PBS, and plated in serial dilutions onto CDMM agar plates containing 50 μg/ml 5′-Fluorcytosine. Emerging colonies were restreaked onto BHI and BHI containing thiamphenicol. Cultures growing on BHI, but not on selective BHI were screened for second recombination events.

Successful manipulation at the *modT* locus was determined by colony PCR using FFO-498/499 and integrity of homology and the knockout region was verified by Sanger sequencing (FFO-498, 499, 482, 483). Similarly, double crossover recombinants at the *pyrE* locus were identified using FFO-612/613 and verified by Sanger sequencing (FFO-610, 611, 612, and 613). For sequencing of the 01583-*dccA* operon knockout, FFO-1743, 1746, and FFO-1749 were used.

### Hot phenol extraction of total RNA and DNase I digestion

For collection, samples were mixed with ice-cold Stop Mix (95% EtOH, 5% acidic phenol) equivalent to 1/5 of the culture volume and subsequently frozen in liquid N_2_. For RNA extraction, samples were thawed on ice, centrifuged for 20 min at 4,000 × g, 4°C. Pellets were suspended in 600 μl of 10 mg/ml lysozyme in TE buffer (pH 8) and digested for 10 min at 37°C, and 60 μl of 10% (w/V) SDS was added, samples were mixed by inversion and incubated at 64°C for 1 to 2 min. Subsequently, 66 μl of 3 M NaOAc, pH 5.2 with 1 mM EDTA were added and mixed by inversion, followed by addition of 750 μl of acidic phenol for RNA extraction (Roth). Samples were mixed by shaking and incubated at 64°C in a water bath for 6 min with intermediate mixing. Phases were separated by centrifuging at 4°C, 16,000 × g for 15 min and the aqueous phase was transferred to a Phase Lock Gel tube (Eppendorf), where an equal volume of chloroform was added. Phases were thoroughly mixed and subsequently separated by centrifugation at 16,000 × g, 15°C for 12 min. Again, the aqueous phase was transferred to a new tube and 2 volumes of a 30:1 Ethanol to 3 M NaOAc (pH 6.5) mix was added and mixed by inversion. RNA was precipitated overnight, centrifuged at 16,000 × g, 4°C for 30 min and washed twice with 500 μl ice cold 75% Ethanol, with 10-min centrifugation steps in between. After complete removal of liquid, the pellet was air dried at RT for approximately 5 min and eluted in 50 μl of RNase free water by shaking at 1,000 RPM, 65°C, for 10 min. RNA concentration and purity was assessed with a Nanodrop 2000 spectrophotometer (Thermo Fisher).

Approximately 5 μg of RNA were digested with 5 U DNase I and 10 U RNase Inhibitor (Takara) at 37°C for 1 h. For RNA purification, the sample was adjusted to 100 μl with DEPC-treated ddH_2_O and transferred to a Phase Lock Gel tube, where it was mixed with 100 μl P:C:I for RNA (Roth) by inversion. Phases were separated by centrifugation at 16,000 × g, 4°C for 15 min. The aqueous phase was transferred to a new tube and precipitated overnight after adding 2.5 volumes of 30:1 Ethanol to NaOAc (pH 6.5) and mixing. Precipitated RNA was centrifuged for 30 min at 16,000 × g, 4°C, washed twice with 200 μl 75% EtOH with 10-min centrifugation steps at 16,000 × g, 4°C, dried for 5 min after complete liquid removal and eluted in 20 μl DEPC-treated water by shaking for 5 min at 1,000 RPM, 65°C.

### Rifampicin assay

To inhibit transcription of growing cultures, Rifampicin was added to *C*. *difficile* WT cells grown in TYG medium to mid-exponential phase, to a final concentration of 200 μg/ml per culture and mixed by swirling. RNA samples were aliquoted at the indicated time points and samples were fixed by adding ice-cold stop mix, inversion and immediate freezing in liquid nitrogen. RNA was extracted as indicated in the hot phenol protocol.

### Northern blotting

For northern blot analysis, 3 to 6 μg of RNA were suspended in 2× Gel loading buffer II (95% formamide, 18 mM EDTA, 0.025% SDS, 2% bromophenol blue), boiled for 5 min at 95°C and subsequently cooled for 5 min on ice. After separation on a 6% polyacrylamide (PAA) gel containing 7 M Urea for 2 h at 300 V, RNA was transferred to a Hybond N+ membrane (GE Healthcare) in 1× TBE using a wet blotting chamber for 1 h at 50 V, 4°C and subsequently cross-linked at 254 nm (0.12 J/cm^2^).

Membranes were pre-hybridized in Roti Hybri-Quick buffer (Roth) for 1 h at 42°C. RNA of interest was detected with [γ-^32^P]-labeled oligonucleotides by overnight hybridization at 42°C. A list of all oligonucleotides used in this study can be found in supplementary **S4 Dataset**. The membrane was washed sequentially in 5×, 1×, and 0.5× SSC containing 0.1% SDS, dried and finally exposed on a phosphor screen for 1 to 7 days. Signals were visualized using a Typhoon FLA7000 phosphor imager. Band intensities were assessed using ImageJ v1.52u [58].

Blots were stripped with 0.1% SDS in distilled, boiled water for 15 min and subsequently washed in distilled water. Blots were hybridized in Roti Hybri-Quick buffer before adding a new oligonucleotide probe.

### Oligonucleotide labeling

For [γ-^32^P] 5′-labeling, 10 pmol of DNA-oligonucleotide probes were incubated with 5 U T4 Polynucleotide Kinase (NEB) together with 1 μl [γ-^32^P]-ATP (10 μCi/μl) in a volume of 10 μl. After 1 h incubation at 37°C, 10 μl of sterile, DNase- and RNase free water was added and radiolabeled oligonucleotides were separated from free [γ-^32^P]-ATP using a sephadex G-25 column (Cytiva) according to the manufacturer’s instructions.

### In vitro transcription of RNA, RNA labeling, and in-line probing (ILP)

ILP assays were performed as described previously [53]. The short and the long isoform of ModT were PCR amplified using FFO-1235/1089 and FFO-1235/1088 to add a T7 promoter. RNA was in vitro transcribed using the T7 Megascript RT Kit (Thermo Scientific), resolved on a 6% PAA, 7 M Urea gel, excised and eluted overnight in 750 μl RNA elution buffer (0.1 M NaOAc, 0.1% SDS, 10 mM EDTA) at 1,000 RPM, 8°C. RNA was purified by adding 750 μl P:C:I for RNA (Roth), thoroughly mixed and phases were separated by centrifugation. The aqueous phase was transferred to a new tube and RNA was precipitated by adding 1 ml 30:1 EtOH:NaOAc (pH 6.5).

Approximately 50 pmol of in vitro transcribed RNAs were dephosphorylated by CIP (NEB) treatment, purified and precipitated as described before and 20 pmol of eluted RNAs were radioactively 5′-labeled using 10 U T4 Polynucleotide kinase (Thermo Scientific) and 2 μl [γ-^32^P]-ATP (10 μCi/μl) in 1× PNK Buffer A (Thermo Scientific) in a total volume of 20 μl for 30 min at 37°C. Labeled RNA was purified using a sephadex G-50 column (Cytiva) and a denaturing PAA gel, isolated by overnight elution from the gel and purified by organic phase separation and precipitation of the aqueous phase as described above.

For ILP reactions, labeled ModT was denatured at 95°C for 1 min and subsequently chilled on ice for 5 min, and 0.04 pmol/μl ModT was incubated in a buffer containing final concentration of 100 mM KCl, 20 mM MgCl_2_, 50 mM Tris-HCl for approximately 40 h at RT in a volume of 15 μl. Control reactions were set up in RNase free water. For a T1 ladder, 0.01 U/μl of RNaseT1 (Thermo Scientific) was incubated with 0.02 pmol/μl of labeled ModT RNA for 5 min at 37°C in a volume of 15 μl. For the alkaline hydrolysis control reaction, 0.02 pmol/μl ModT was heated to 95°C for 5 min in sodium bicarbonate (alkaline hydrolysis) buffer, pH 9 (Thermo Scientific). All reactions were stopped by adding an equal volume of 2× colorless loading dye (10 M Urea, 1.5 mM EDTA). Finally, 10 μl of the quenched reactions was separated on 60-cm long denaturing PAA gels at 40 W, which were dried onto whatman paper for 2 h at 80°C. The radioactive signal was exposed to a phosphor screen, which was read out with a Typhoon FLA 7000 imager (Fuji series).

### DMS-structure probing and sequencing

#### In vitro DMS-structure probing

For in vitro dimethyl sulfate structure probing (DMS-structure probing), a PCR template was amplified from *C*. *difficile* 630 gDNA using a high-fidelity Fusion DNA-polymerase (Mobidiag) with the primers od1192 and od1193 for the long ModT isoforms, and od1192 and od1194 for the short ModT isoform, appending a T7 promoter sequence to the template.

PCR templates were used for in vitro transcription reactions as described above, which were subsequently purified over a silica column and eluted in RNase free water. RNA size and integrity was verified on a 7 M Urea 4% PAA gel.

The RNA stock was denatured for 2 min at 95°C and subsequently chilled on ice for 5 min, and 50 ng of each RNA transcript was allowed to fold for 15 min at 37°C in a “physiological buffer” (20 mM HEPES-NaOH (pH 7.5), 140 mM KCl, 2 mM MgCl_2_). DMS diluted in EtOH was added to the folded RNAs (final concentration 85 mM) and incubated at 37°C for 6 min. For each condition, an EtOH-only control was included. Reactions were quenched on ice by adding 200 mM final of β-mercaptoethanol (Sigma-Aldrich) and 3 volumes of TRI reagent LS (Sigma Aldrich).

RNA was extracted according to the manufacturer’s instuctions. Briefly, 0.2 volumes of chloroform was added, followed by thorough mixing. Samples were centrifuged at 12,000 × g for 15 min, 4°C and the aqueous phase was transferred to a new tube in which the RNA was precipitated by adding 1 volume of isopropanol and 1 μl of Glycoblue (Invitrogen) followed by a 30 min incubation at −80°C. RNA was centrifuged at 12,000 × g, 4°C for 30 min and the pellets were washed twice with 1 ml of 75% EtOH, air dried, and eluted in 15 μl water.

For cDNA synthesis, half the volume of RNA (7.5 μl) was heated to 65°C together with 1.5 μl 100 μm primer (LongModT_Rv for the long ModT isoform and ShortModT_Rv for short ModT isoform) for 5 min and used for reverse transcription with the Marathon-RT [59] in RT-buffer (50 mM Tris-HCl (pH 8.3), 200 mM KCl, 5 mM DTT, 20% glycerol, 1 mM MnCl2, 0.5 mM dNTP, and 8 U RNaseIn) for 8 h at 42°C.

cDNAs were diluted to 1/8 with nuclease-free water, and 5 μl the diluted cDNA was used as template in 50 μl PCR reaction using the Q5 High-Fidelity DNA Polymerase (NEB) and the primers od1195 and od1194 for the long ModT isoform, and od1195 and od1197 for the short ModT isoform, appending Nextera sequencing adapter sequences HF and HR to the amplicons extremities. Cycling conditions were 2 min at 98°C then 25 cycles of 10 s at 98°C, 15 s at 55°C, and 15 s at 72°C then 72°C for 5 min, and 5 μl of PCR products were verified on a 1.5% agarose gel and the rest was column purified using NucleoSpin Gel and PCR Clean-up kits, Macherey-Nagel according to the manufacturer’s instructions.

A final indexing PCR was carried out using Illumina Nextera DNA CD indexes (96 Indexes, 96 Samples, Illumina). Reaction conditions were 80 ng of purified PCR product, 1 × Q5 reaction buffer, 0.2 mM dNTPs, 2.5 μl of indexing primer, 0.02 U/μl of Q5 polymerase in a final volume of 14 μl. Indexed PCR products were verified on a 1.5% agarose gel, pooled together in an equimolar ratio, before final purification on a 1.5% agarose gel. The pooled indexed sequencing library was quantified using the NEBNext library Quant Kit for Illumina and paired-end PE150 sequencing was carried out on an Illumina Novaseq instrument (Novogene). DMS-MaP data was trimmed using cutadapt v 1.18 [59] and aligned to the reference sequence using bowtie2 [60]. cutadapt parameters were “–nextseq-trim 25 –maxn 0 -g gtgatatttaggtctgtg -G gatatttagctgacctgg.” bowtie2 parameters were “-D 20 -R 3 -N 1 -L 15 -i S,1,0.50.” Further analysis was carried out using the rf-count and rfnorm modules of RNA Framework package v2.7.2 [61]. rfcount parameters were “—count-mutations–only-mut G>Y;A>B;C>D;T>V -q 30” and a sample specific mask file was included to ignore counting at primer binding sites. rf-norm parameters were “-rb AC -sm 3 -nm 2 –remap-reactivities,” meaning that DMS reactivities were calculated by subtracting background mutations in the untreated sample and normalized using 90% Winsorizing.

The match between predicted RNA structures and DMS reactivities was carried out using python v 3.12.3 with the sklearn.metrics.roc_auc_score function from the scikit-learn v 1.5.1 package.

#### In vivo DMS-structure probing

*C*. *difficile* 630 WT cultures were inoculated in a time-matched manner, so that the structure probing procedure was carried out at the same time for all cultures. For native DMS-probing analysis, 3 ml (~2 to 5 ODs) of culture were treated with 150 μl of 3.4 M DMS in EtOH (final concentration 170 mM) or an equal volume of EtOH for 6 min at 37°C. The reaction was quenched by adding 150 μl of β-mercaptoethanol 14 M (Sigma-Aldrich) and transferring cells on ice. Cells were centrifuged at 5,000 × g for 10 min, 4°C and the pellet was dissolved in 1 ml of TRI reagent (Sigma Aldrich). To support lysis, the ice-cold samples were transferred into tubes containing 500 mg of glass beads and subjected to 3 grinding cycles (30 s, 6 m/s) in a FastPrep (MP Biomedicals) beadbeater with cooling on ice for 2 min between each cycle. Samples were centrifuged for 10 min at 10,000 × g, 4°C. The bead-free supernatant was transferred to a new tube and RNA was extracted according to the manufacturer’s instructions, and eluted in 15 μl RNase free water.

For deproteinated lysate DMS-probing analysis, 6 ml of culture (~2 to 5 ODs) was centrifuged at 5,000 × g for 15 min, 4°C and the bacterial pellet was dissolved in 1 ml of lysis buffer (20 mM Tris-HCl (pH 7.5), 150 mM KCl, 1 mM MgCl2, 1 mM DTT, 1 mM PMSF, 0.2% Triton ×100, 200 U/ml RNaseIn). Cells were lysed mechanically at 4°C, and the lysate was clarified by centrifugation 5 min at 10.000 × *g*, 4°C; 110 μl of SDS 10% and 50 μl of proteinase K (NEB) were added to 1 ml of clear lysate and the mixture was incubated at 37°C for 30 min with gentle shaking. The digested lysate was equally distributed in 2 microtubes, one added with 50 μl of DMS (85 mM in EtOH) and one added with 50 μl of EtOH, and the incubation was prolonged at 37°C for 6 min. The reaction was quenched by adding 50 μl of β-mercaptoethanol 14 M and transfer on ice, and 1.5 ml of TRI reagent LS was added and the RNA was extracted according to the manufacturer’s instructions, and eluted in 15 μl RNase free water.

Approximately 8 μg of each probed RNA sample (native or deproteinated) were treated with 2 μl Turbo DNase (Thermo Scientific) with 5 μl 10× Turbo DNase buffer and 12 U RNaseIn in a total volume of 50 μl for 30 min at 37°C and subsequently purified over a silica column with NTC buffer (Macherey-Nagel); 1 μg DNase-digested total RNA was used for cDNA synthesis using the Marathon-RT with the conditions indicated above (using the primer LongModT_Rv for the long ModT isoform specifically and ShortModT_Rv for the short ModT isoform, which includes ModT long). cDNAs were purified over a silica column with NTC buffer (Macherey-Nagel). Half of the purified cDNA was PCR-amplified with the Q5 High-Fidelity DNA Polymerase using HF_ModT_Fw and HR_ShortModT_Rv as previously described. PCR products were analyzed on a 1.5% agarose gel and purified on silica column (Macherey-Nagel). Indexing PCR, library preparation, and data analysis was carried out as described in the previous section.

### Reverse transcription and quantitative PCR

For cDNA strand synthesis, 1 μg of DNase I digested RNA was reverse transcribed using the M-MLV reverse transcriptase Kit (Invitrogen) with random hexamer primers (Invitrogen) according to the manufacturer’s instructions.

qPCR reactions were performed using 1 μl of 20-fold diluted cDNA and with 2x Takyon NO ROX SYBR Master Mix blue TTP (Eurogentec) with 20 nM primers in a total volume of 10 μl. Each reaction was carried out at least in technical duplicates per biological replicate. Fold change was calculated with the 2^-ΔΔCT^ method [62] using 16S rRNA as a housekeeping reference.

### RNA sequencing

Samples for RNA sequencing were collected from *C*. *difficile* WT and Δ*modT* cultures grown in TYG medium to EP (3, 5h) and to TP (9 h). RNA was extracted using the hot phenol method, DNase I digested and purified as indicated above. cDNA library preparation and sequencing were performed by Vertis Biotechnologie AG. RNA samples were examined by capillary electrophoresis. Ribosomal RNA was depleted using an in-house developed protocol. The ribodepleted RNA samples were fragmented using ultrasound (1 pulse of 30 s at 4°C) and an oligonucleotide adapter was ligated to the 3′ end of the RNA molecules. First-strand cDNA synthesis was performed using M-MLV reverse transcriptase and the 3′ adapter as primer. The first-strand cDNA was purified and the 5′ Illumina TruSeq sequencing adapter was ligated to the 3′ end of the antisense cDNA and the resulting cDNA was PCR-amplified (15 cycles) to about 10 to 20 ng/μl using a high fidelity DNA polymerase. The cDNA was purified using the Agencourt AMPure XP kit (Beckman Coulter Genomics) and analyzed by capillary electrophoresis to estimate library quality and concentration. For Illumina NextSeq sequencing, the samples were pooled in approximately equimolar amounts. The cDNA pool was size fractionated in the size range of 200 to 600 bp using a preparative agarose gel. An aliquot of the size fractionated pool was analyzed by capillary electrophoresis for quality control. The cDNA pool was single-read sequenced on an Illumina NextSeq 500 system using 75 bp read length.

### Bioinformatic analysis of RNA sequencing

Raw reads of RNA-seq libraries were trimmed and filtered using BBDuk (ktrim = r, k = 23, mink = 11, hdist = 1, tpe, tbo, qtrim = r, trimq = 20, minlength = 25). Mapping and read counting was conducted with READemption 0.4.3 (READemption align:—poly_a_clipping,—realign, READemption gene_quanti:—skip_antisense,—no_count_splitting_by_gene_no) [63]. Reads were mapped to *C*. *difficile* 630 CP010905.2 with additional annotations of UTRs and ncRNAs. Differential expression analysis was performed with edgeR [64]. Normalization factors were calculated using the trimmed means of M values method (TMM). All features with less than 10 reads in at least 2 libraries were excluded from the analysis.

### Fluorescence quantification by flow cytometry

At the respective time point, 200 μl of each sample was transferred to a 96-well plate and centrifuged at 4,500 × g, 4°C for 10 min. Supernatant was removed using a multichannel pipette and bacterial pellets were suspended in 150 μl of 4% PFA and fixed at RT in a dark plate for 30 min. Bacteria were washed 3 times in 200 μl PBS with centrifuging steps as indicated above. The final cell pellet was suspended in 30 μl PBS containing 1 μg/ml DAPI and kept overnight in the dark at 4°C to allow full fluorophore maturation. The next day, samples were centrifuged, supernatant was removed and pellets were thoroughly suspended in 200 μl of PBS. Cells were diluted to an appropriate concentration and red fluorescence (λ_ex_ = 561 nm, signal detection with the custom “PE-Texas Red” bandwidth filter detecting 615 +/−20 nm) was determined using a Novocyte Quanteon flow cytometer (Agilent) from 100,000 events, selecting exclusively DAPI-positive cells (λ_ex_ = 405 nm, applying the “Pacific-Blue” filter (detecting 445 +/−45 nm) and a FSC-H (>5,000 AU) cutoff to discriminate cells from false positive debris events. The mean fluorescence of the selected events was taken as readout. To normalize fluorescence, the mean fluorescence from the inactivated riboswitch (pRS^A70G^) was subtracted from the functional, c-di-GMP sensitive reporter (pRS) in each respective background. Fluorescence of all strains carrying the nonfluorescent control (pEV) was measured, but was not included in the calculation.

## Supporting information

S1 Fig Analysis of ModT RNA features. **(A)** Read profiles obtained from differential RNA-seq (dRNA-seq) and RNAtag-seq libraries. For dRNA-seq, half of the libraries are treated with terminator exonuclease (TEX+) which degrades processed (non-triphosphorylated) 5′ ends. This differential treatment results in a relative read enrichment for primary transcripts (5′-PPP) in the TEX+ libraries, whereas it results in a relative read enrichment for processed transcripts in the TEX- libraries. As a result, it allows the identification of transcript start sites (TSS) and 5′ processing sites. For RNAtag-seq, adapters for sequencing are ligated to RNA 3′ ends, which leads to enriched read coverage at 3′ ends of transcripts, thus facilitating the annotation of transcription termination sites (TTS). The genome coordnates correspond to genome annotation CP010905.2. The data underlying this figure can be found in https://doi.org/10.1073/pnas.2103579118 and under the accession number GSE155167. **(B)** ModT expression profile in *C*. *difficile* 630 across different growth stages. Total RNA was extracted from *C*. *difficile* 630 grown in TY medium supplemented with indicated carbon sources at indicated time points (same RNA as in Fig 1C). ModT transcript was detected by northern blot using a radioactively labeled DNA probe (FFO-358) specific for the extended 3′ end of the long ModT isoform (ModT(L)). 5S rRNA served as loading control. **(C)** Quantification of RNA half-lives based on 3 biological replicates, as displayed in Fig 1D. The calculated half-life of each transcript is shown above. The underlying data can be found in S5 Dataset. **(D)** GC content of noncoding RNA elements. Analysis was based on a manually curated genome annotation (CP010905.2) for *C*. *difficile* 630 that includes annotation of noncoding gene loci [26]. The dotted line indicates the average GC content of the genome. The underlying data can be found in S5 Dataset.
(TIF)

S2 Fig In vitro and in vivo DMS structure probing of ModT isoforms. **(A–D)** In-line probing reactions (ILP) of in vitro transcribed, 5′ ^32^P-labeled ModT(S) and ModT(L) isoforms, resolved on denaturing (7 M Urea) 10% PAA gels (**A, B**). In parallel, the same samples were resolved on a 6% PAA-7M Urea gel (**C, D**) to increase resolution of residues in the 3′ end of both isoforms. RNA was left untreated as control (Ctr). Denatured RNA, digested by RNase T1 (T1) or cleaved by alkaline hydrolysis (OH), served as ladder. Base-paired regions are indicated according to the secondary structure model shown in Fig 2A. **(E)** Heatmap displaying correlations of all DMS reactivity samples calculated using the Pearson’s correlation coefficient and hierarchical clustering using the single linkage algorithm. **(F)** The Δreactivity values for each nucleotide position obtained for the indicated conditions were plotted onto the *C*. *difficile* ModT consensus secondary structure model shown in Fig 2A–2C. For “in vivo Native → Δ(3h-9h),” the profiles of ModT(L) and ModT(S) were combined to obtain the mean reactivity and standard deviation at these 2 time points. For “ModT(L) → Δ(Deproteinated–Native),” the mean subtracted reactivity and its standard deviation was calculated from all growth stages (3 h, 9 h, and 24 h). **(G)** Heatmap displaying ROC-AUC scores demonstrating the fit of DMS reactivities from each sample to every structure model depicted in Fig 2A–2C. An ROC-AUC score of 0.5 signifies a random association of reactivity and base pairing status, and 1 indicates the perfect agreement.
(TIF)

S3 Fig ModT regulates bacterial transition into the stationary phase. **(A)** qPCR quantification of expression levels of ModT as well as immediate up- and downstream located genes in wild-type and Δ*modT* during exponential phase (EP, 3, 5 h) and transition phase (TP, 9 h). Unpaired *t* test with Welch correction. The underlying data can be found in S5 Dataset. **(B)** Northern blot-based expression analysis of ModT in WT, Δ*modT* and complemented strains across different growth stages in TYG medium. 5S rRNA served as loading control. Depicted is a representative of 3 biological replicates. **(C)** Maximum optical density (OD) of wild type and Δ*modT* grown in TY medium supplemented with indicated carbon sources (*n* = 3 biological replicates). Significance was determined with an unpaired *t* test with Welch correction. The underlying data can be found in S5 Dataset. **(D)** Growth curves of *C*. *difficile* wild type and Δ*modT* indicating the time points of RNA isolation for sequencing. EP = exponential phase, 3, 5 h; TP = transition phase, 9 h. The underlying data can be found in S5 Dataset. **(E)** Venn diagram displaying overlap of up- and down-regulated genes in Δ*modT* relative to wild type between exponential phase (EP, 3, 5 h) and transition phase (TP, 9 h). **(F)** Venn diagram displaying overlap of the SigB regulon [36] (log_2_FC≥1, *p* ≤ 0.05) and differentially expressed genes in Δ*modT* during exponential phase (EP, 3, 5 h) and transition phase (TP, 9 h) (log2FC ≥1/≤-1, FDR < 0.1). Numbers of up- or down-regulated transcripts in Δ*modT* are depicted below. **(G)** Northern blot-based expression analysis of ModT, tRNA His, tRNA Ala, and 5S RNA in wild type and Δ*modT* across different growth stages in TYG medium. Depicted is a representative of 3 biological replicates.
(TIF)

S4 Fig CFU-based quantification of sporulation frequencies. **(A)** and **(B)** Sporulation frequency in 70:30 medium determined by plate-based assay. Total CFU of spores germinated on BHIS-agar supplemented with 0.1% Taurocholate (top) and of vegetative bacterial cells recovered on BHIS agar (bottom) are displayed in (A). Calculated sporulation frequency based on CFU values (A) is displayed in (B). Significance for data shown in (A) and (B) was determined by two-way ANOVA with Tukey’s multiple comparisons test. Only significant differences with *P* < 0.05 are displayed [**P < 0.01, ***P < 0.001, ****P < 0.0001]. The underlying data can be found in S5 Dataset. **(C)** Northern blot-based expression analysis of ModT in WT, Δ*modT* and complemented strains across different growth stages in 70:30 sporulation medium. 5S rRNA serves as loading control. Depicted is a representative of 3 biological replicates.
(TIF)

S5 Fig ModT modulates cellular c-di-GMP levels. **(A)** Schematic representation of riboswitch reporter. Green box represents the constitutive fdxA (CDIF630_00294)-promoter, the double stem loop with black pin represents the repressing c-di-GMP II riboswitch conformation at low c-di-GMP levels, while the dashed, white pin indicates the open conformation at high c-di-GMP levels, allowing transcription of the downstream mCherry fluorophore. **(B)** Raw data used for c-di-GMP quantification in Fig 4A. To account for potential variation in promoter activity in the reporter (*p*RS), the signal from the construct with inactivated Riboswitch (*p*RS^A>G^) was subtracted from the actual reporter pRS. In addition, an empty vector control (*p*EV) was used to normalize for potential differences in baseline fluorescence due to different mutant backgrounds. *n* = 3 biological replicates per strain. Significance was determined by two-way ANOVA with Tukey’s multiple comparisons test. Only significant differences with *P* < 0.05 are displayed [***P < 0.001, ****P < 0.0001]. The underlying data can be found in S5 Dataset.
(TIF)

S6 Fig Orthologs from distant phylogenetic families can replace ModT function in *C*. *difficile*. **(A)** Sequence alignment of ModT orthologs from *C*. *difficile* (*Cd)*, *P*. *sordellii* (*Ps*), *C*. *perfringens* (*Cp*), *Acetohalobium arabaticum (Aa)*, and *Clostridium* sp. CAG:138 (CAG). Color-coding of regions P1-P8 is identical to Fig 2A. **(B)** Schematic representation of chromosomal *modT* deletion and replacement of the short ModT isoform in *C*. *difficile* 630 with orthologous sequences from *P*. *sordellii* (Ps), *C*. *perfringens* (Cp), *Acetohalobium arabaticum* (Aa), and *Clostridium* sp. CAG:138 (CAG). **(C)** Northern blot-based expression analysis of ModT in WT, Δ*modT* and in *C*. *difficile* 630 with ModT orthologue replacements across different growth stages in 70:30 medium. 5S rRNA serves as loading control. Depicted is a representative of 3 biological replicates. (**D**) Representative phase-contrast micrographs of cultures used for calculating sporulation frequencies in **Fig 6C**. Scale bar = 5 μm. **(E)** Plate-based sporulation assay in 70:30 medium for WT, Δ*modT* and *C*. *difficile* 630 with ModT orthologue replacements. Total CFU of vegetative bacterial cells growing on BHIS agar and total CFU of germinated spores determined in BHIS-agar supplemented with 0.1% Taurocholate. Time points are indicated relative to the onset of the transition phase. Experiments were performed in (*n* = 3) biological replicates. Significance was determined by two-way ANOVA with Dunnett’s multiple comparisons test. Only significant differences with *P* < 0.05 are displayed [**P < 0.01, ***P < 0.001, ****P < 0.0001]. The underlying data can be found in S5 Dataset. **(F)** Calculated sporulation frequency based on CFU and spore values shown in (**E**). Significance was determined using two-way ANOVA with Dunnett’s multiple comparisons test. Only significant differences with *P* < 0.05 are displayed [**P < 0.01, ***P < 0.001, ****P < 0.0001]. The underlying data can be found in S5 Dataset. **(G)** Alignment of promoter region upstream of the transcription start site of *modT* in *C*. *difficile* (*Cd)*, *P*. *sordellii* (*Ps*), *C*. *perfringens* (*Cp*), and *C*. *acetobutylicum* (*Ca*).
(TIF)

S1 Dataset RNA Seq of total RNA from *C*. *difficile* 630, as published before [26]. Annotated transcript products are sorted by their normalized expression indicated as reads per kilobase per million mapped reads (RPKM).
(XLSX)

S2 Dataset Differential expression analysis for Δ*modT* and WT cultures grown in TYG medium to exponential phase (EP, 3, 5 h) and to transition phase (TP, 9 h). (XLSX)

S3 Dataset SigB vs. RNAseq: Differentially expressed genes (DEGs) in the modT mutant that were also up-regulated after sigB induction (log2FC bigger or equal to 1) [35]. Sporulation vs. RNAseq: Differentially expressed genes (DEGs) in the modT mutant that are part of the sporulation pathway. # Indicates genes that are induced during sporulation by Spo0A, σF, σE, σG, or σK [39].
(XLSX)

S4 Dataset Overview of strains, plasmids, oligonucleotides, and gene products used in this study. (XLSX)

S5 Dataset All numerical data underlying figures within this manuscript. (XLSX)

S1 Raw Images All original and uncropped minimally adjusted images of RNA gels and northern blots shown within the manuscript. (PDF)

## Abbreviations

BHI: brain heart infusion
DMS-MaP: dimethyl sulfate mutational profiling
EP: exponential phase
FDR: false discovery rate
ILP: in-line probing
LB: Luria–Bertani
ncRNA: noncoding RNA
OD: optical density
OLE: Ornate Large Extremophilic
PAS: Per-Arnt-Sim
PBS: phosphate-buffered saline
ROC-AUC: receiver operator characteristic–area under the curve
RPM: revolutions per minute
TP: transition phase
TSS: transcriptional start site
WT: wild type
