# Supplementary figures and images for: The conserved noncoding RNA ModT coordinates growth and virulence in Clostridioides difficile
Source: PLoS Biol. 2024 Dec 13;22(12):e3002948. doi: 10.1371/journal.pbio.3002948 (PMC11706538; doi:10.1371/journal.pbio.3002948)

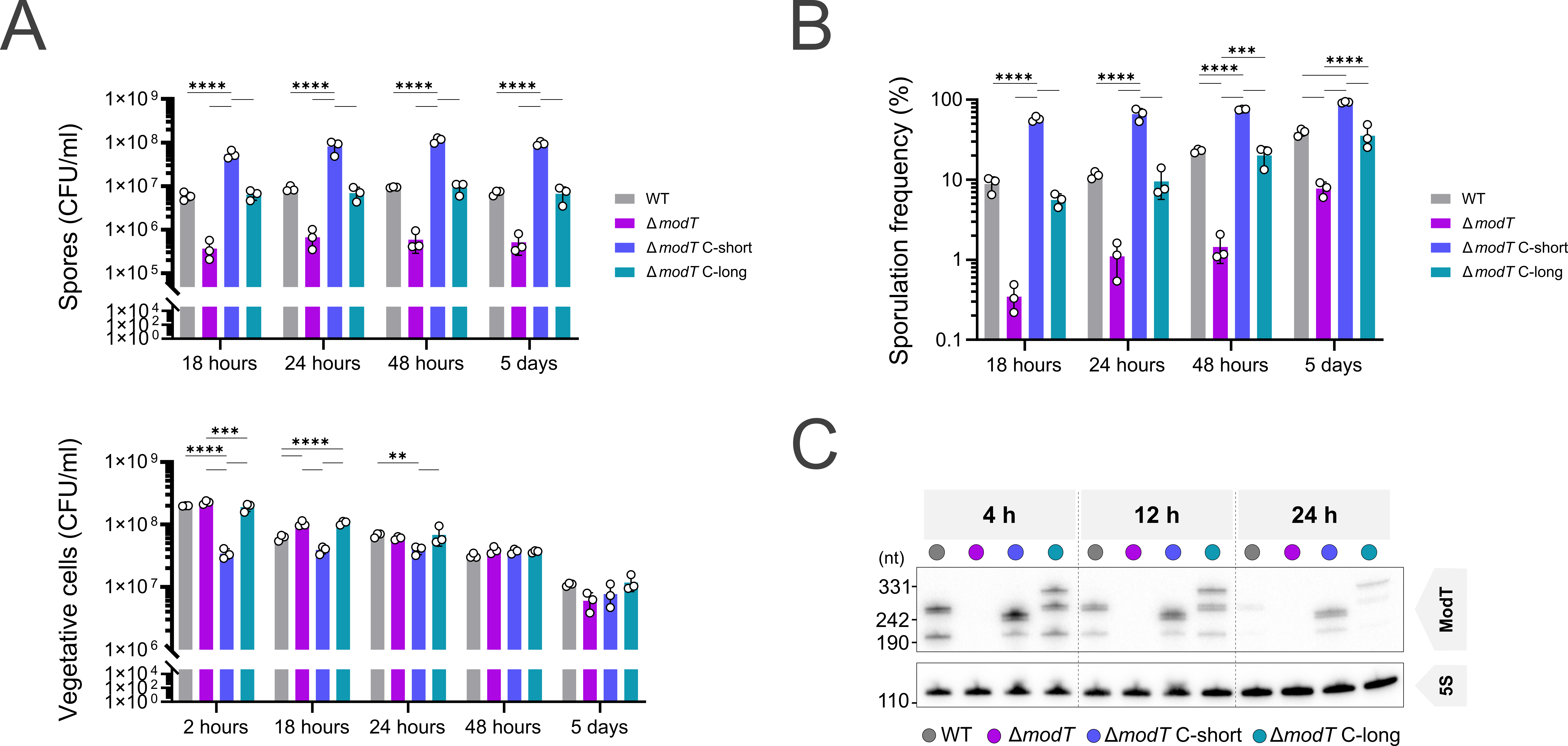

Supplement: S4 Fig — (A) and (B) Sporulation frequency in 70:30 medium determined by plate-based assay. Total CFU of spores germinated on BHIS-agar supplemented with 0.1% Taurocholate (top) and of vegetative bacterial cells recovered on BHIS agar (bottom) are displayed in (A). Calculated sporulation frequency based on CFU values (A) is displayed in (B). Significance for data shown in (A) and (B) was determined by two-way ANOVA with Tukey’s multiple comparisons test. Only significant differences with P < 0.05 are displayed [**P < 0.01, ***P < 0.001, ****P < 0.0001]. The underlying data can be found in S5 Dataset. (C) Northern blot-based expression analysis of ModT in WT, ΔmodT and complemented strains across different growth stages in 70:30 sporulation medium. 5S rRNA serves as loading control. Depicted is a representative of 3 biological replicates. (TIF) [file pbio.3002948.s004.tif]

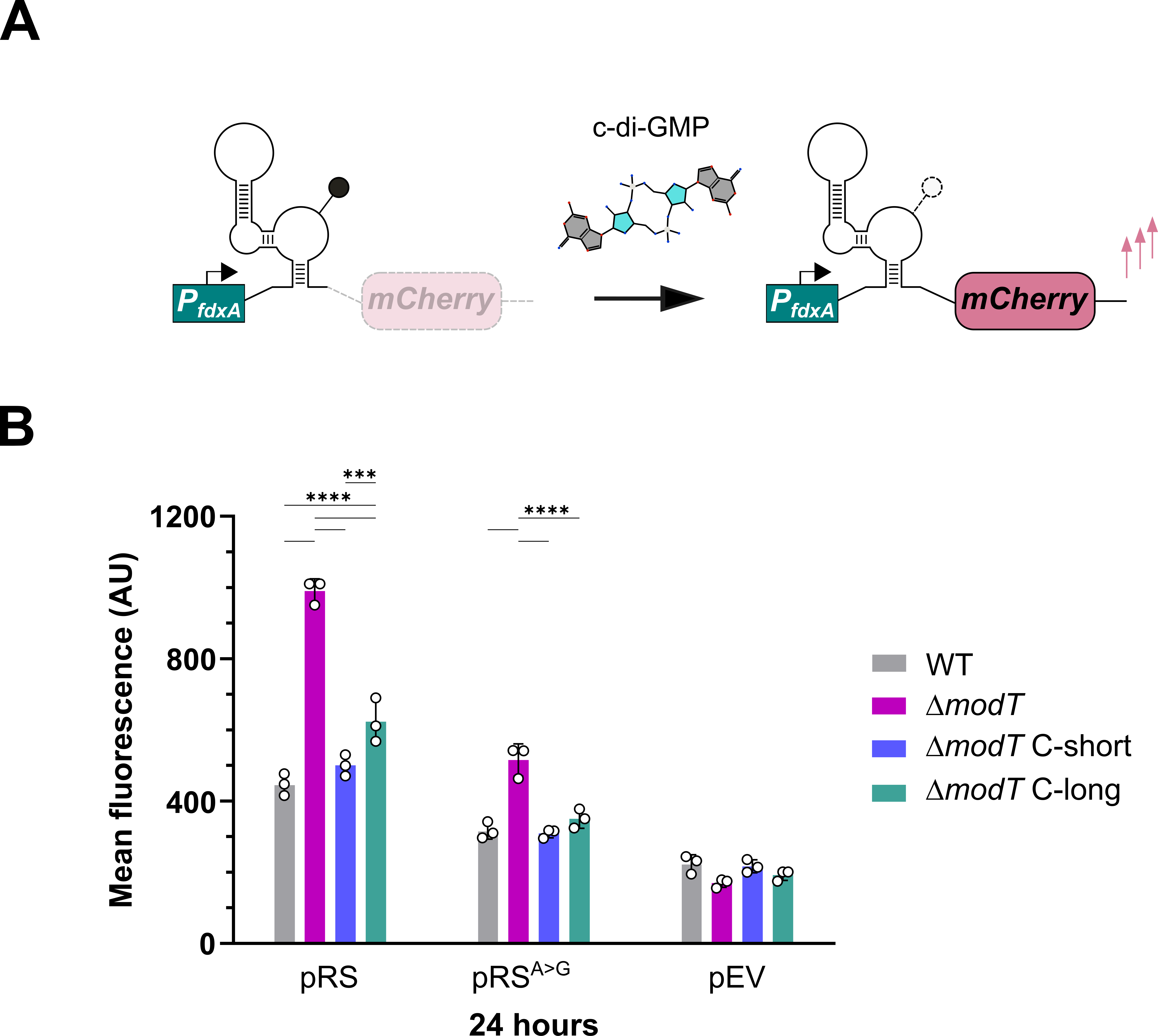

Supplement: S5 Fig — (A) Schematic representation of riboswitch reporter. Green box represents the constitutive fdxA (CDIF630_00294)-promoter, the double stem loop with black pin represents the repressing c-di-GMP II riboswitch conformation at low c-di-GMP levels, while the dashed, white pin indicates the open conformation at high c-di-GMP levels, allowing transcription of the downstream mCherry fluorophore. (B) Raw data used for c-di-GMP quantification in Fig 4A. To account for potential variation in promoter activity in the reporter (pRS), the signal from the construct with inactivated Riboswitch (pRSA>G) was subtracted from the actual reporter pRS. In addition, an empty vector control (pEV) was used to normalize for potential differences in baseline fluorescence due to different mutant backgrounds. n = 3 biological replicates per strain. Significance was determined by two-way ANOVA with Tukey’s multiple comparisons test. Only significant differences with P < 0.05 are displayed [***P < 0.001, ****P < 0.0001]. The underlying data can be found in S5 Dataset. (TIF) [file pbio.3002948.s005.tif]
