# Supplementary material for: The conserved noncoding RNA ModT coordinates growth and virulence in Clostridioides difficile
Source: PLoS Biol. 2024 Dec 13;22(12):e3002948. doi: 10.1371/journal.pbio.3002948 (PMC11706538; doi:10.1371/journal.pbio.3002948)
Supplement: S1 Raw Images — (PDF) [file pbio.3002948.s012.pdf]

Figure 1B

ModT (FFO-67)

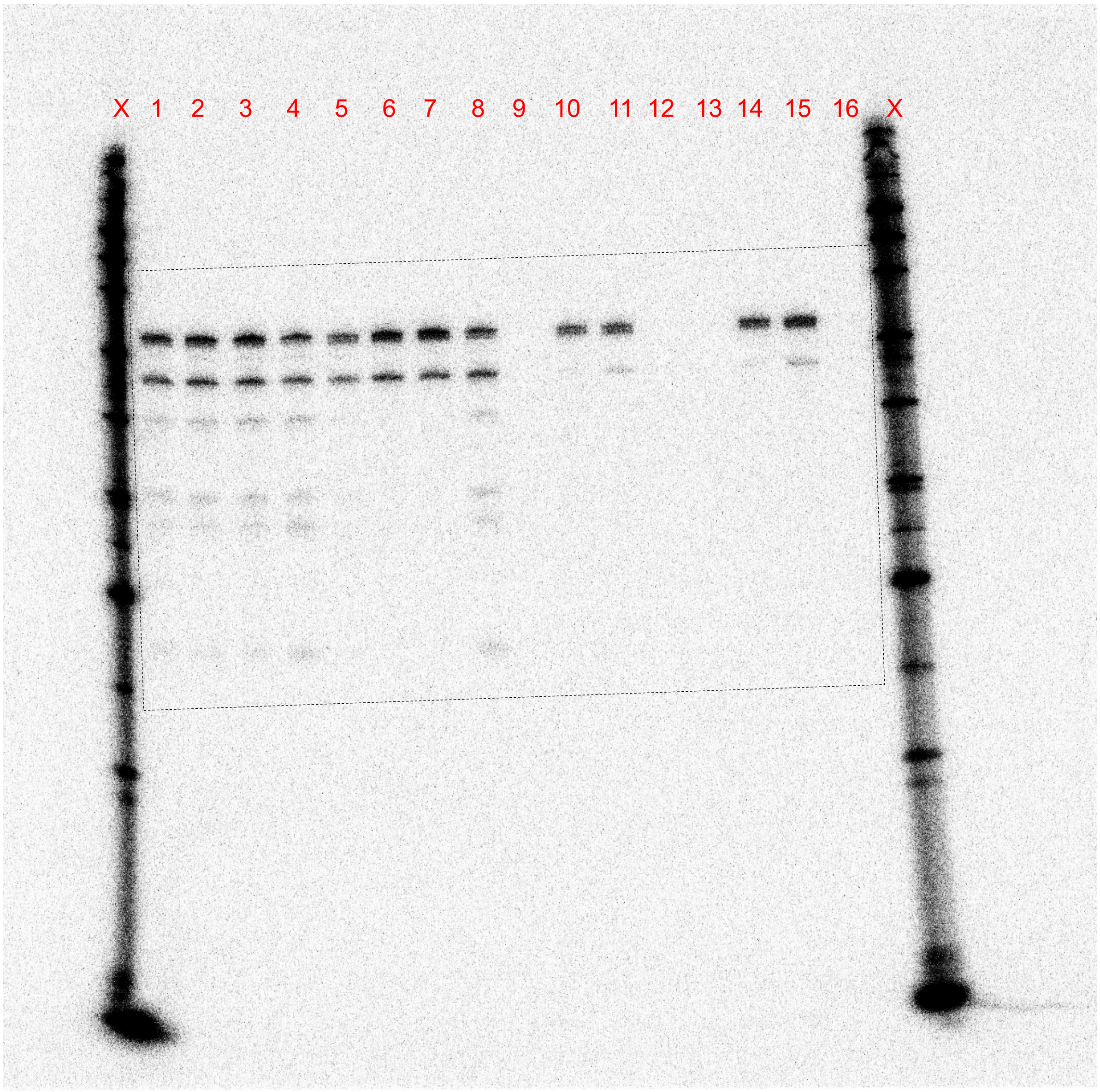

Figure 1B

Probing of blot for 5s rRNA (CD-76) is displayed in source data for Figure S1B

Figure 1D

ModT (FFO-67)

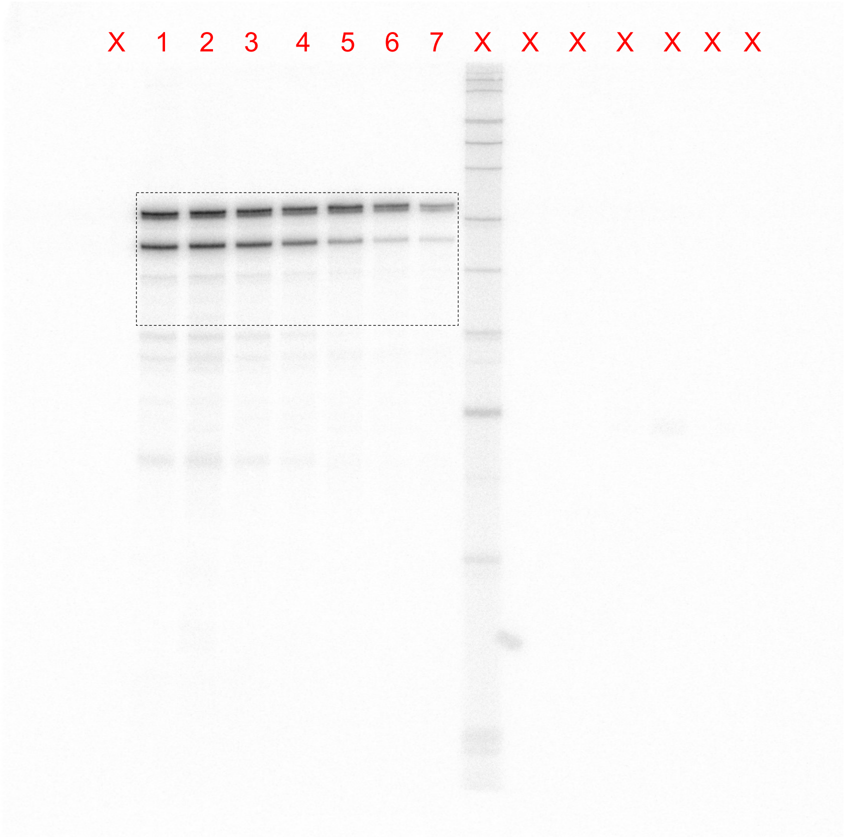

Figure 1D

CDIF630nc_008 (FFO-69)

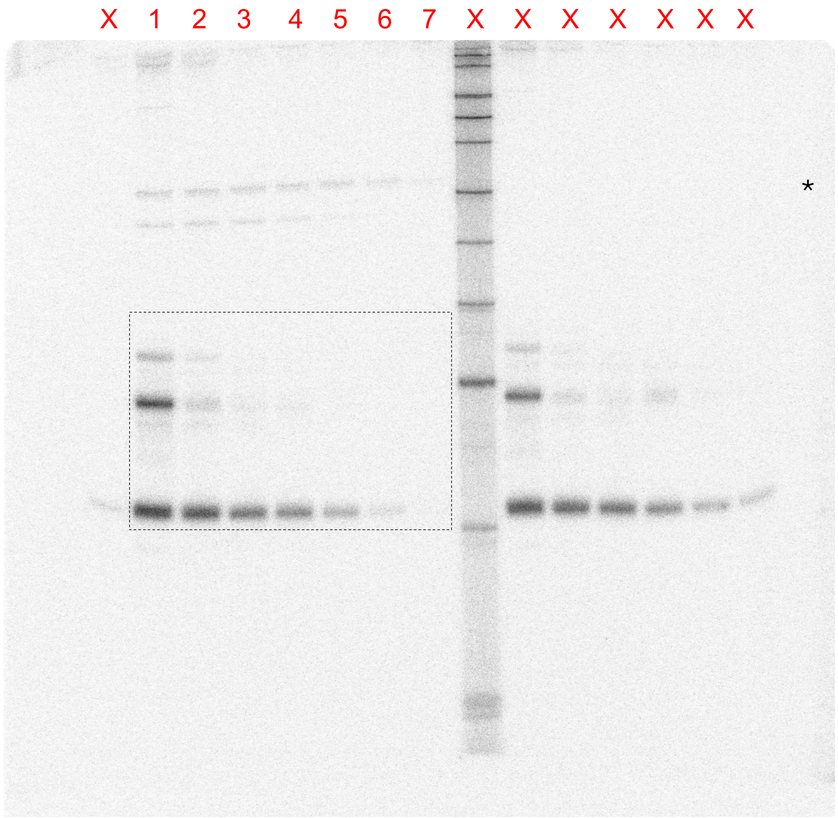

* residual signal from previous probing for ModT (FFO-67)

Figure 1D

5s rRNA (CD-76)

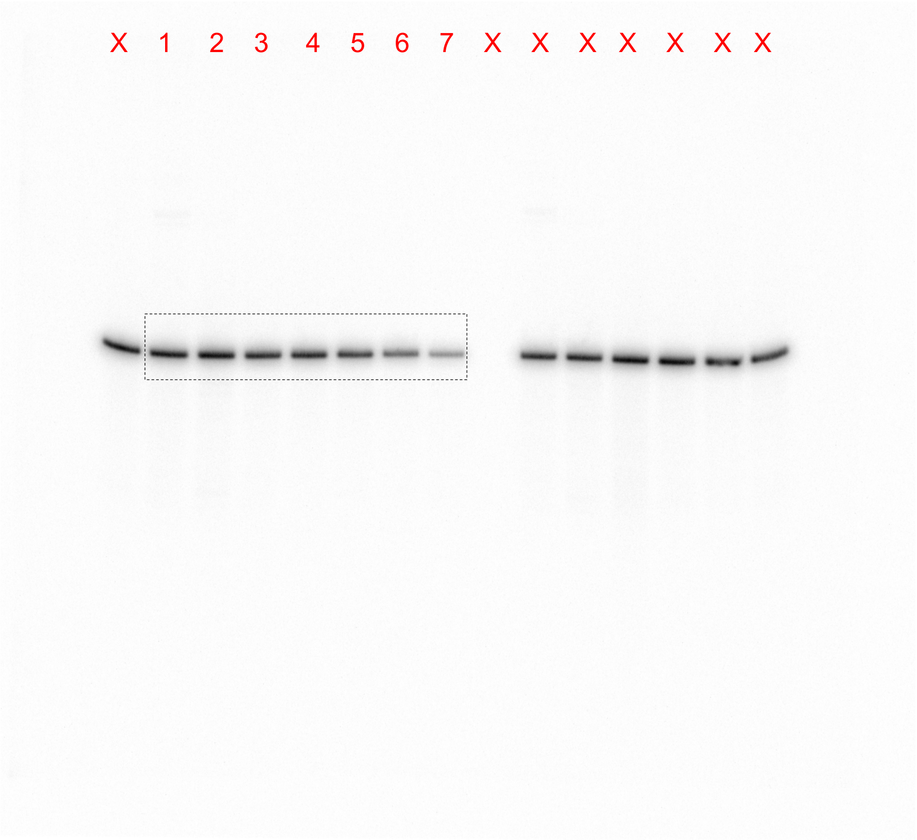

Figure 6D

C. perfringens ModT (FFO-1298)

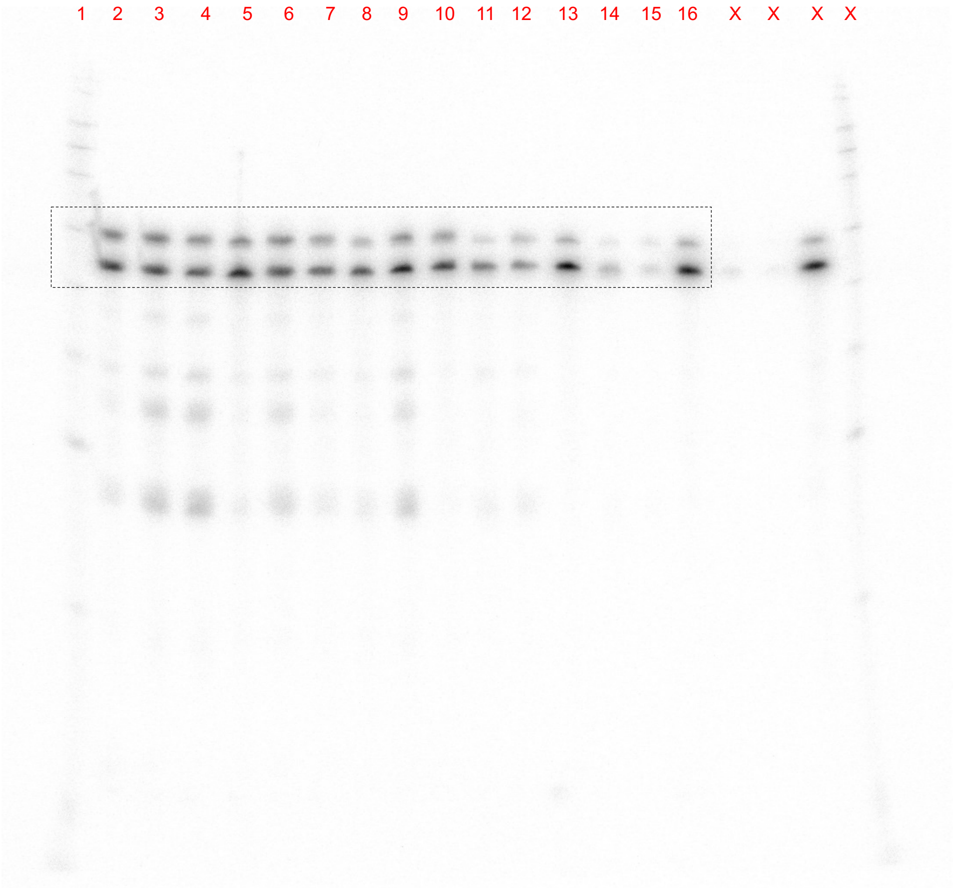

Figure 6D

C. perfringens 5s rRNA (FFO-1320)

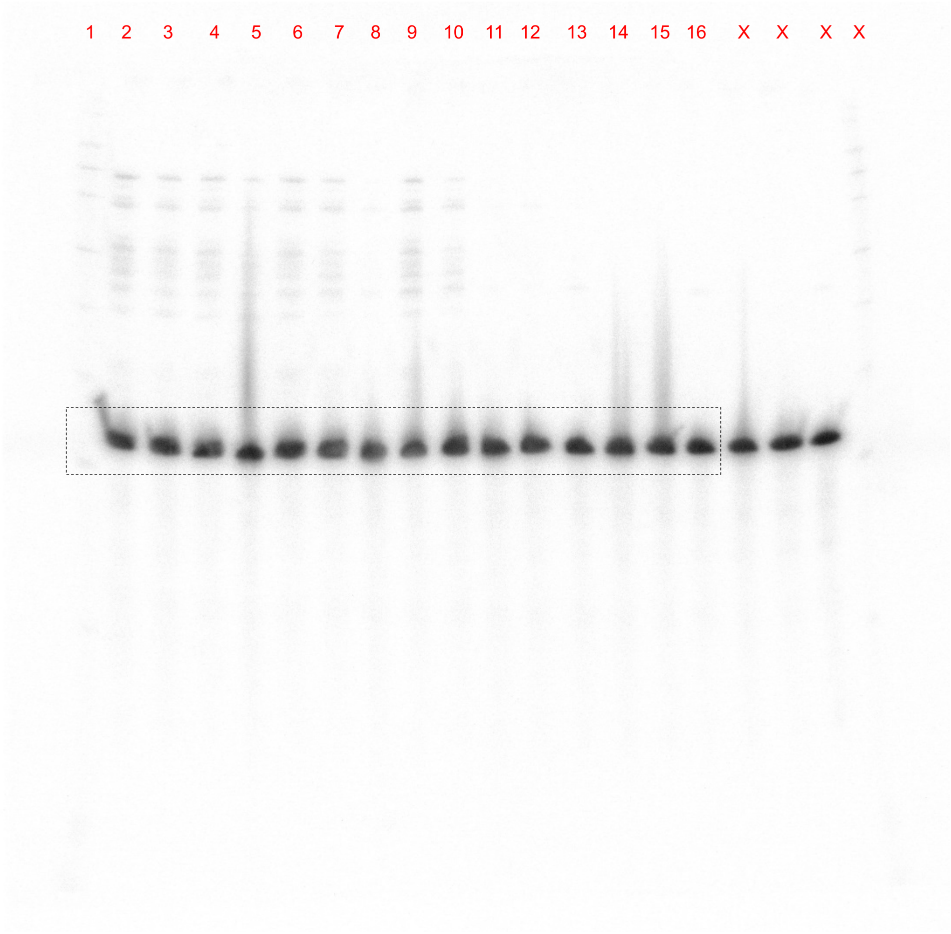

Figure 6D

P. sordellii ModT (FFO-1441)

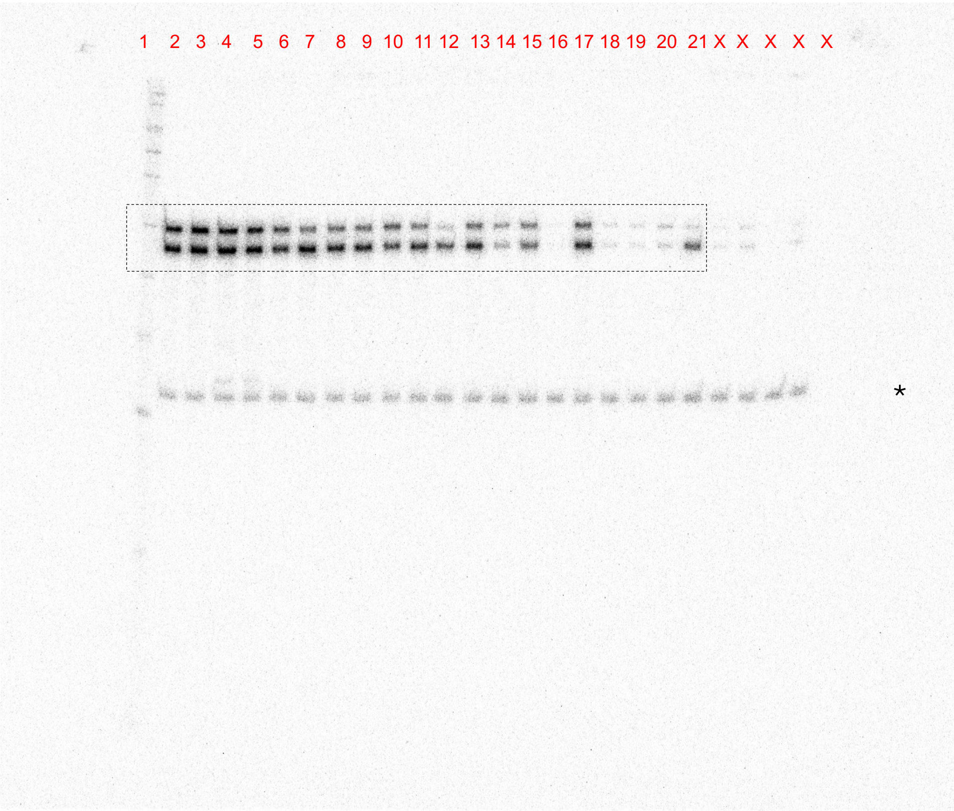

* residual signal from previous probing for 5s rRNA (FFO-1321)

Figure 6D

P. sordellii 5s rRNA (FFO-1321)

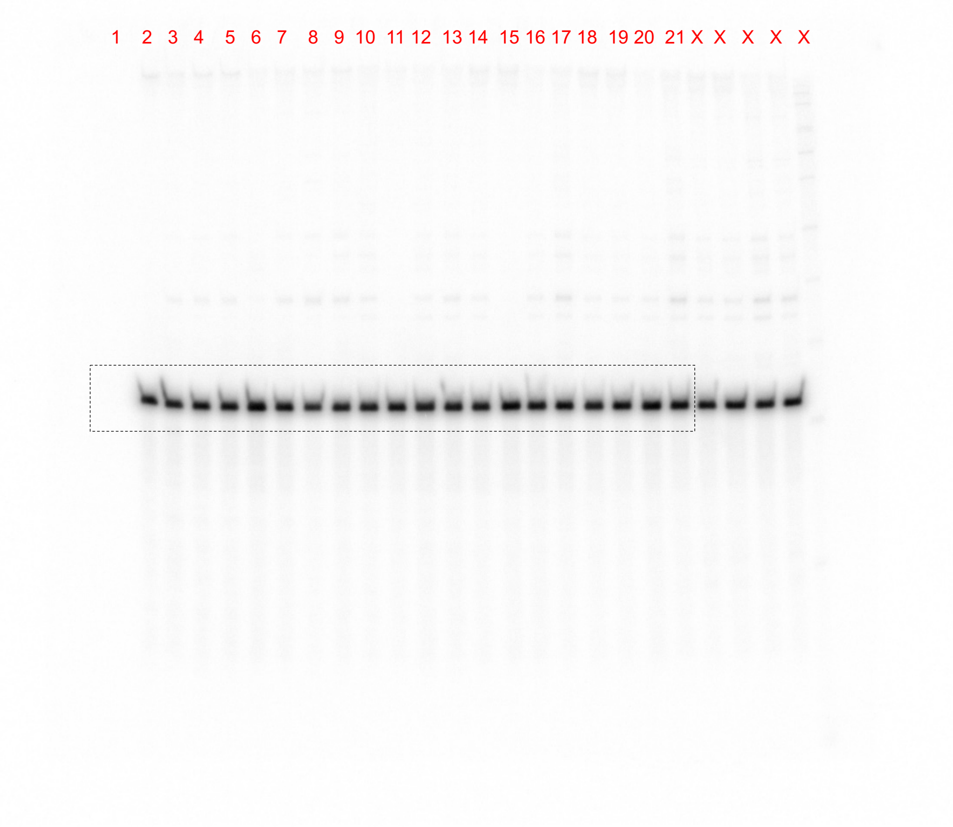

Figure S1B

ModT long (FFO-358)

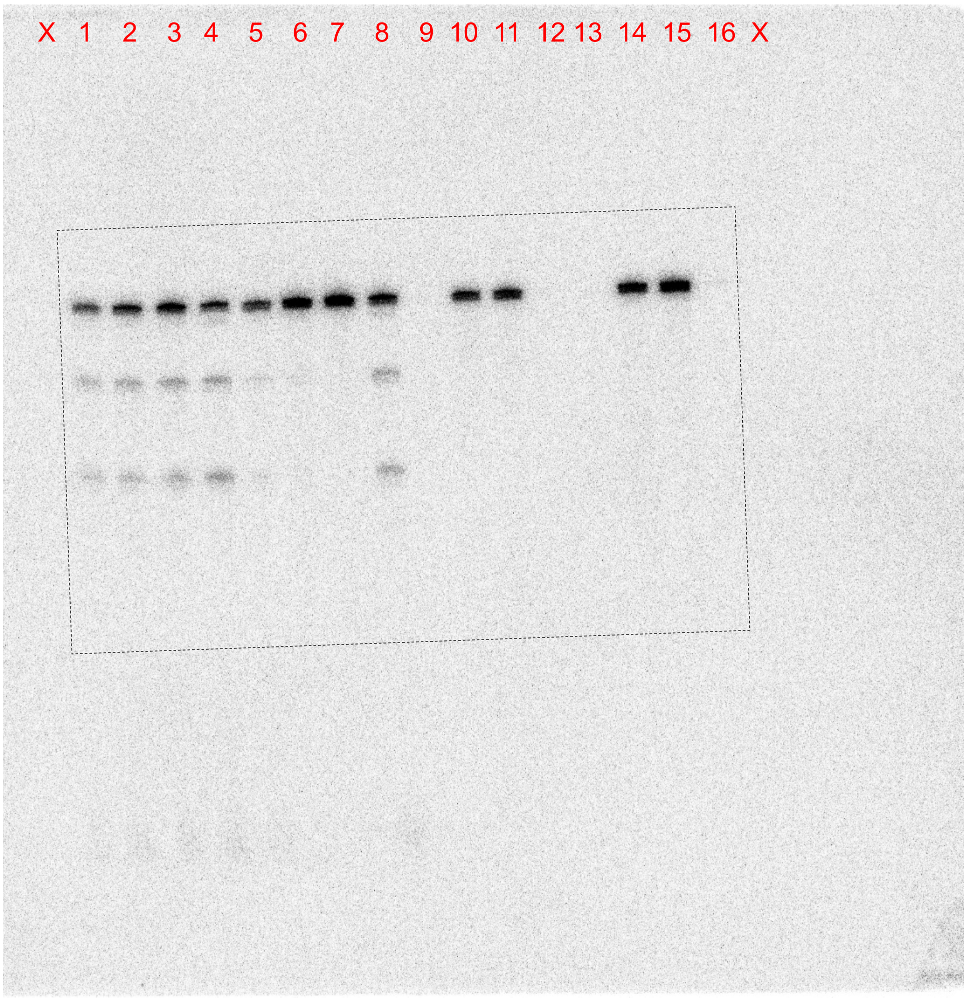

Figure S1B

5s rRNA (CD-76)

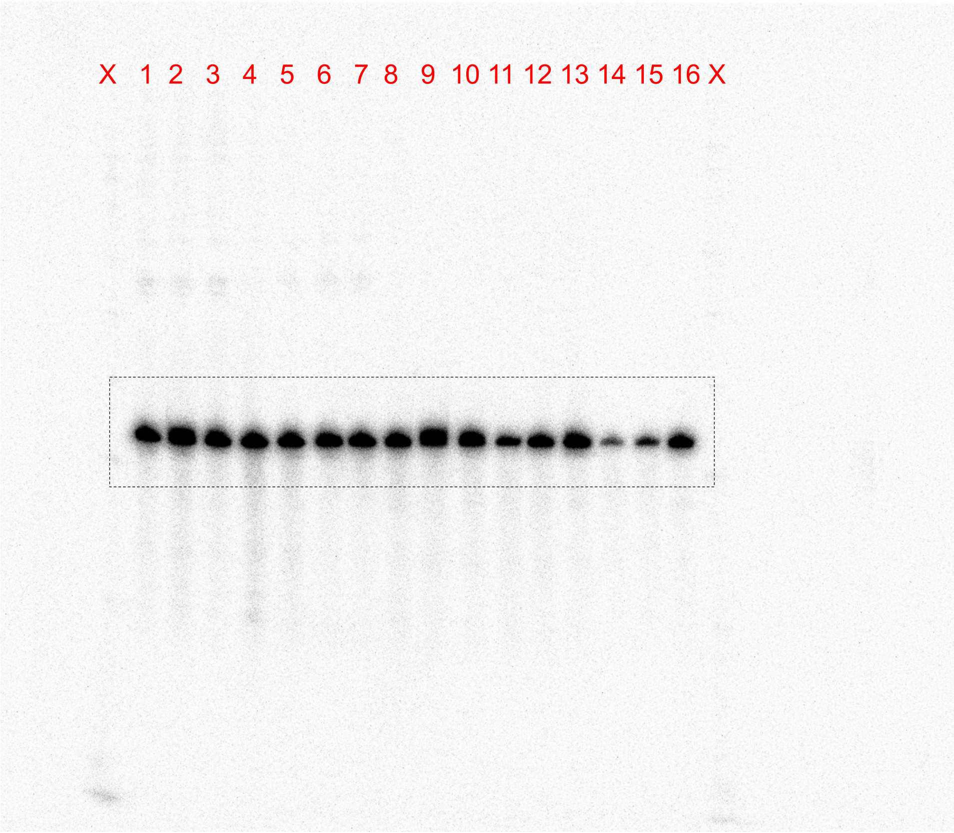

Figure S1C

ModT replicate 2 used for calculation is displayed in Figure 1D.

ModT (FFO-67)
Replicate 1

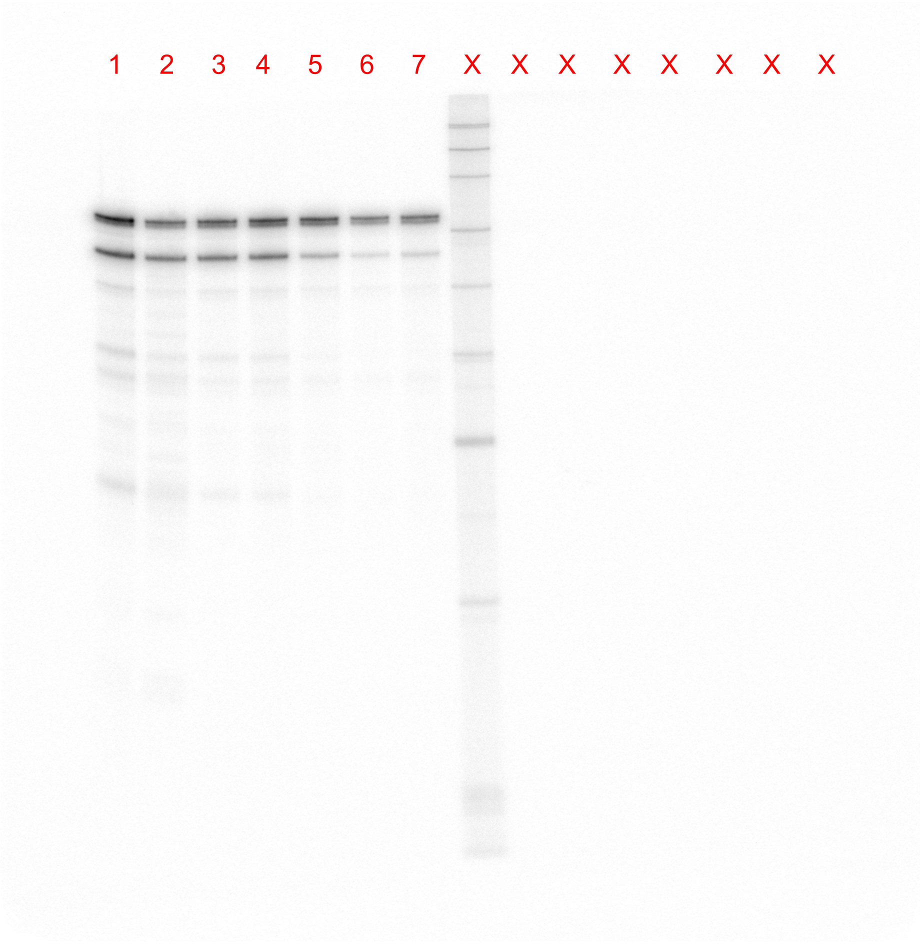

Figure S1C

ModT (FFO-67)

Replicate 3

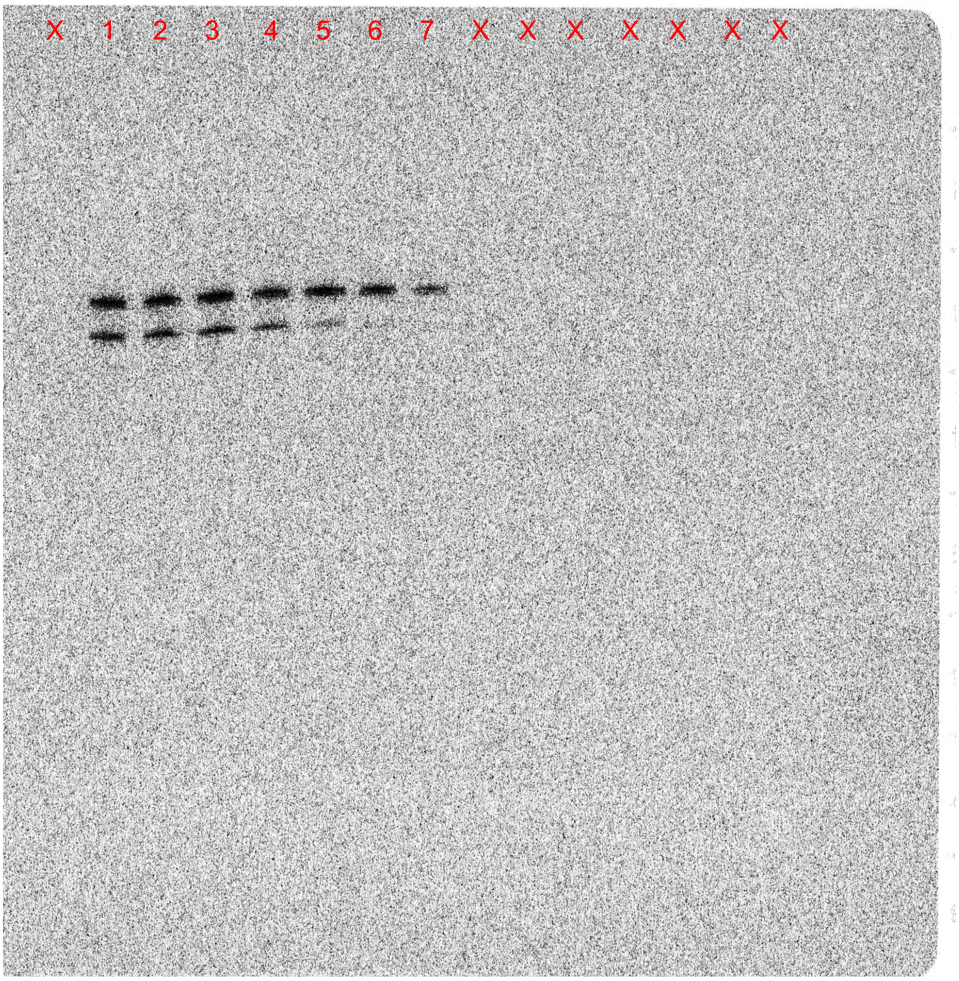

Figure S1C

CDIF630nc_008 replicate 2 used for quantification is displayed in Figure 1D

CDIF630nc_008 (FFO-69)
Replicate 1

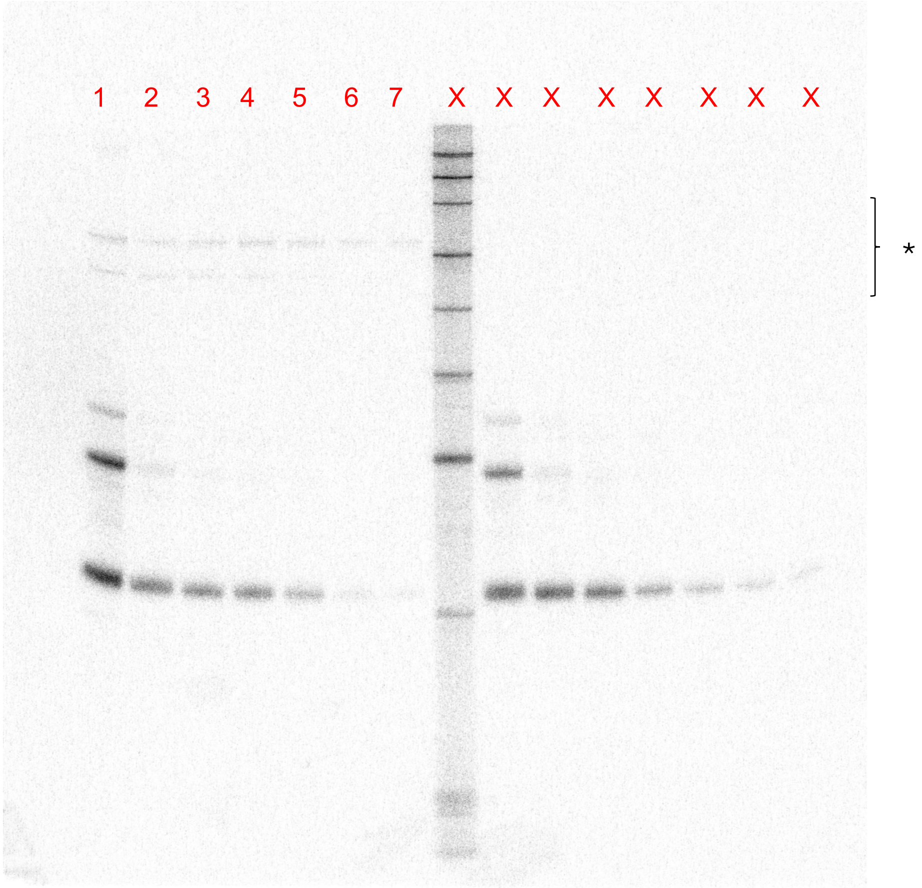

* residual signal from previous probing for ModT (FFO-67)

Figure S1C

CDIF630nc_008 (FFO-69)
Replicate 3

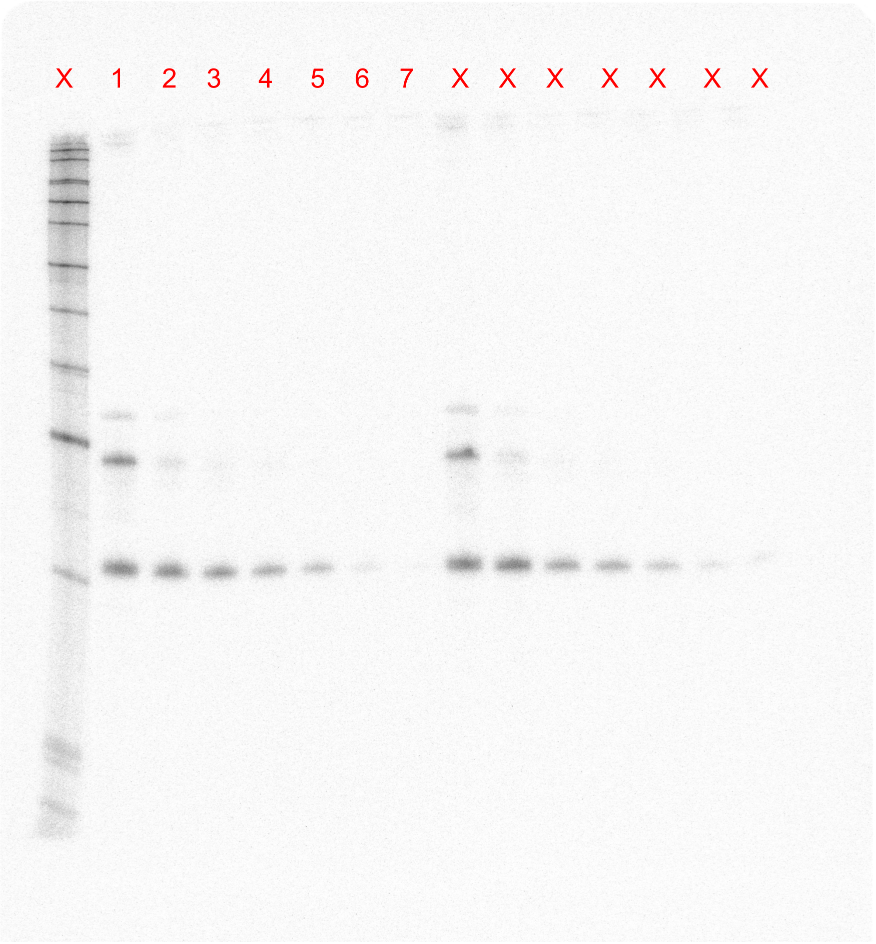

Figure S1C

5s rRNA replicate 2 is displayed in source data for Figure 1D

5s rRNA (CD-76)
Replicate 1

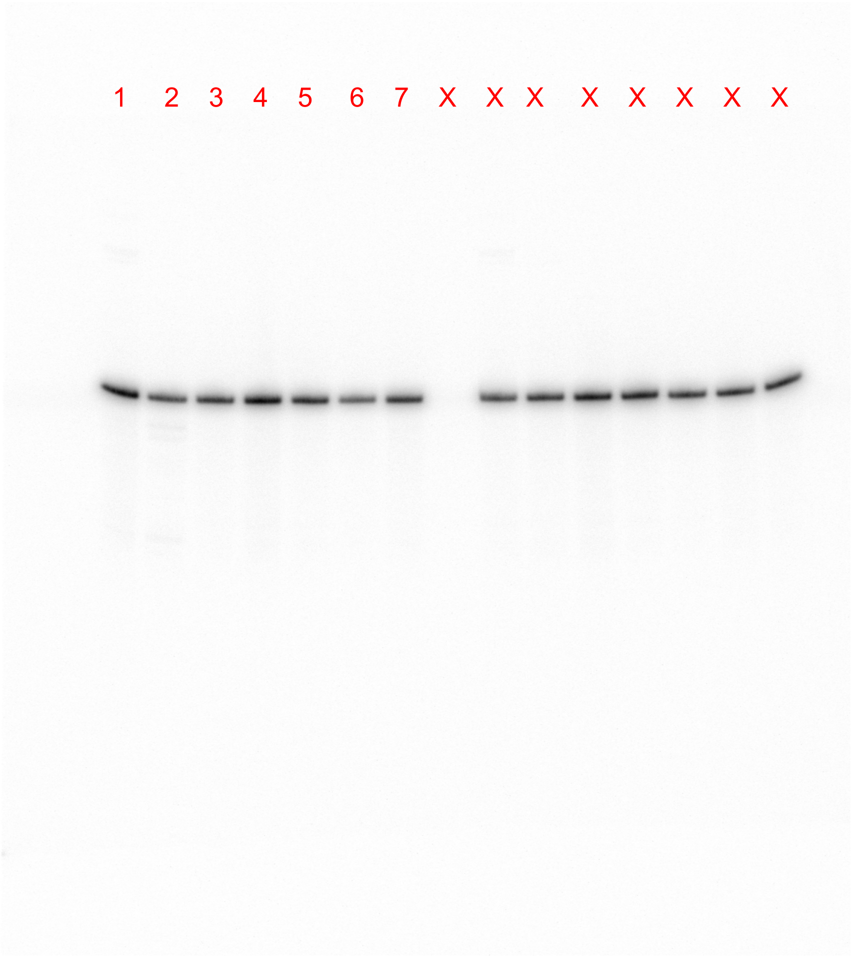

Figure S1C

5s rRNA (CD-76)
Replicate 3

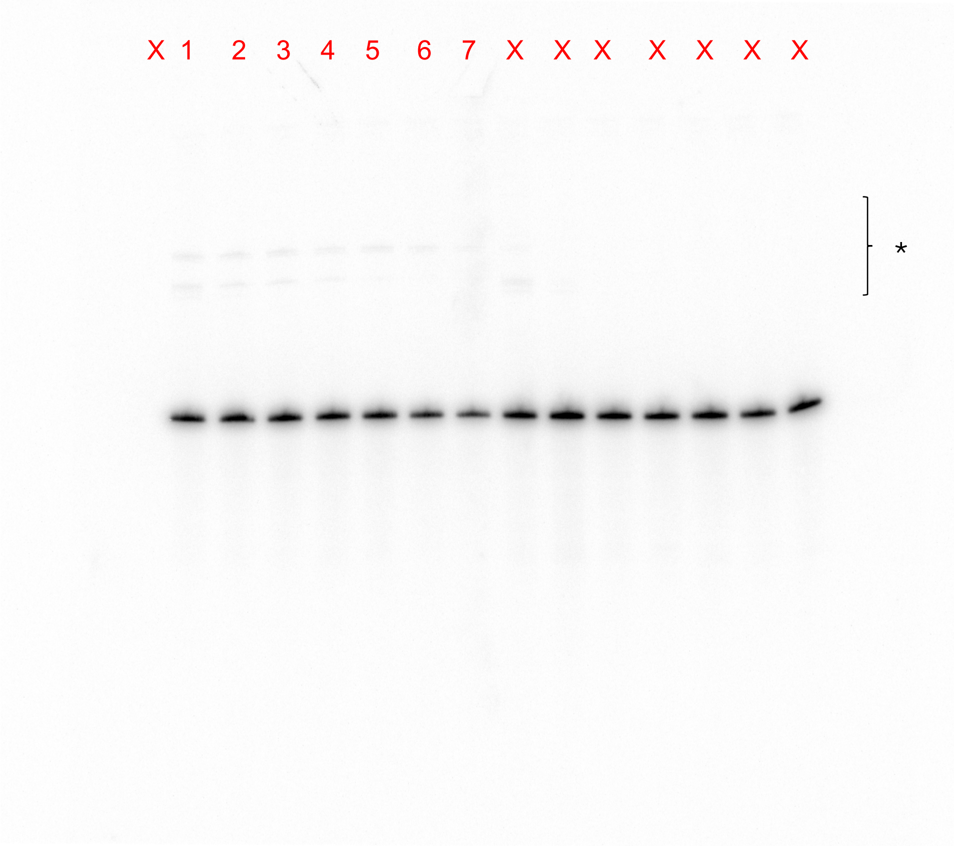

* residual signal from previous probing for ModT (FFO-67)

Figure S2A

ModT short

10% PAA

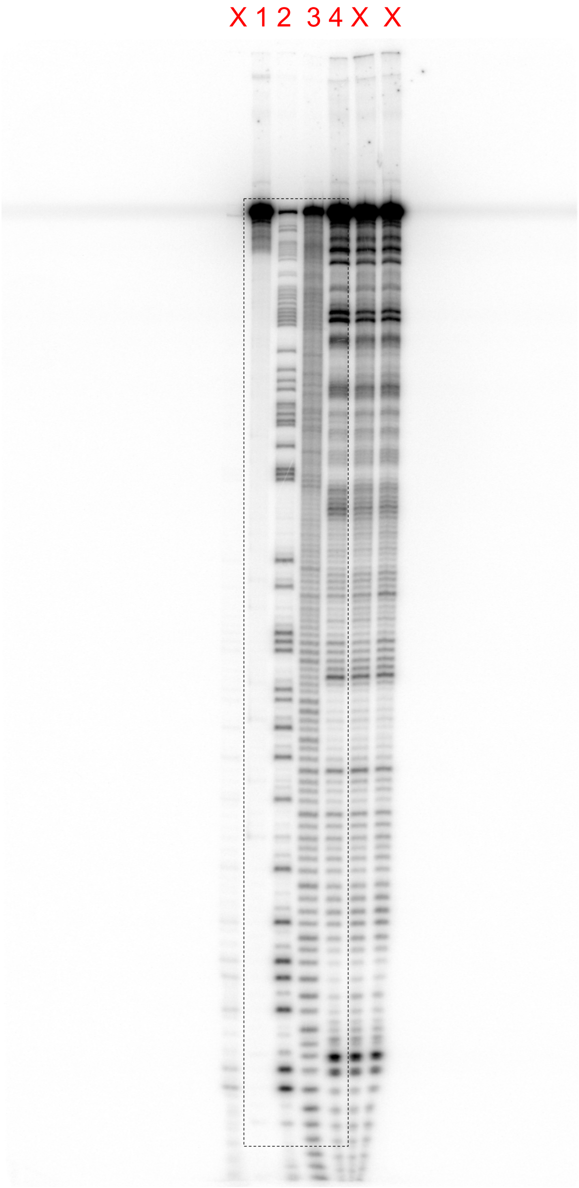

Figure S2B

ModT long

10% PAA

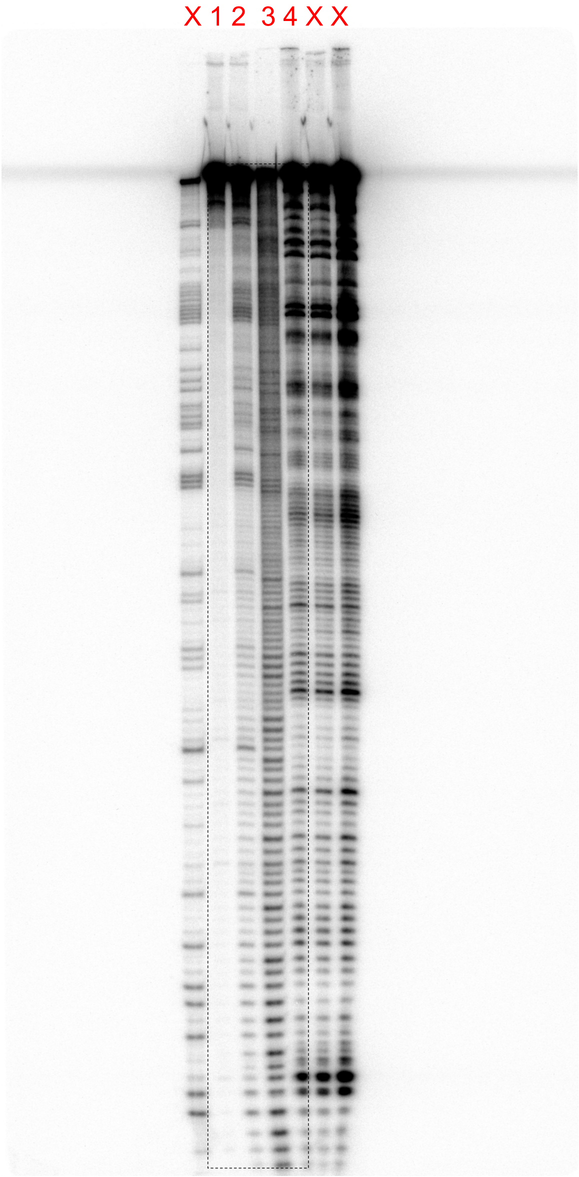

Figure S2C

ModT short

6% PAA

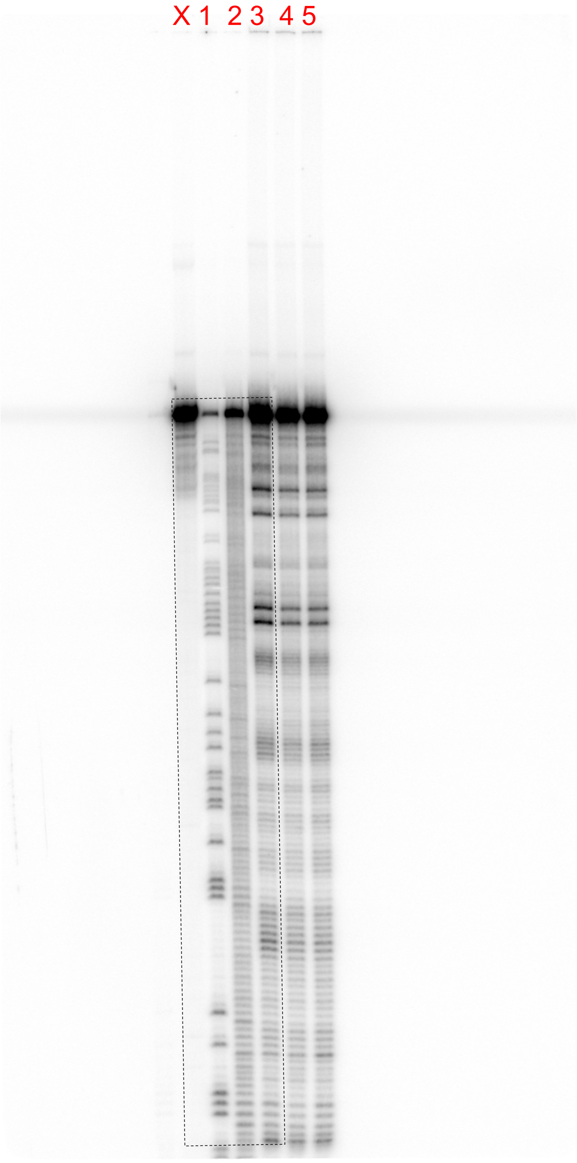

Figure S2D

ModT long

6% PAA

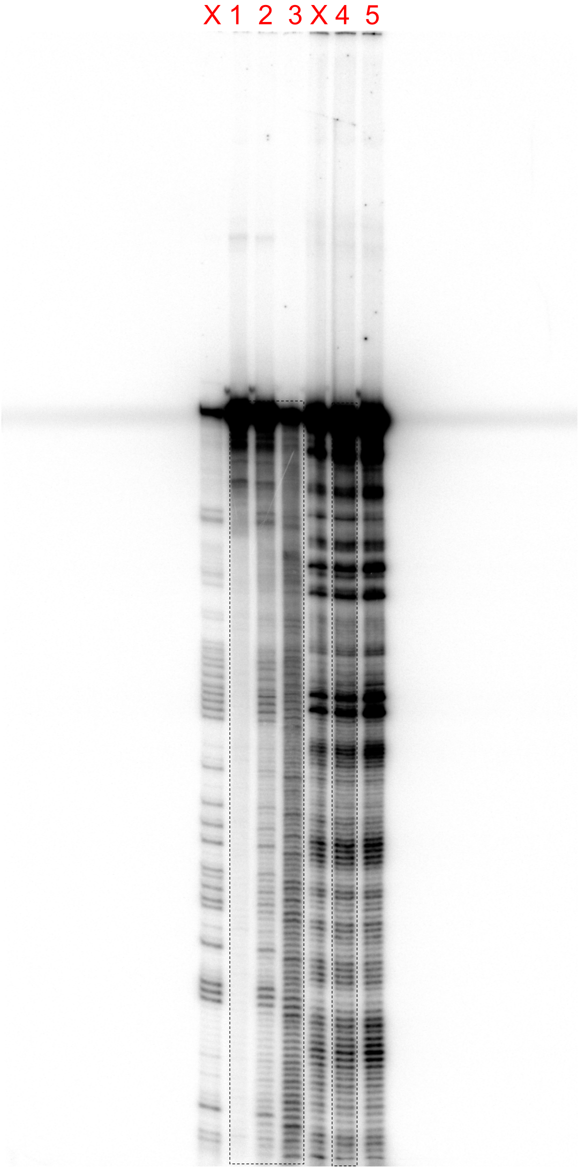

Figure S3C

ModT (FFO-67)

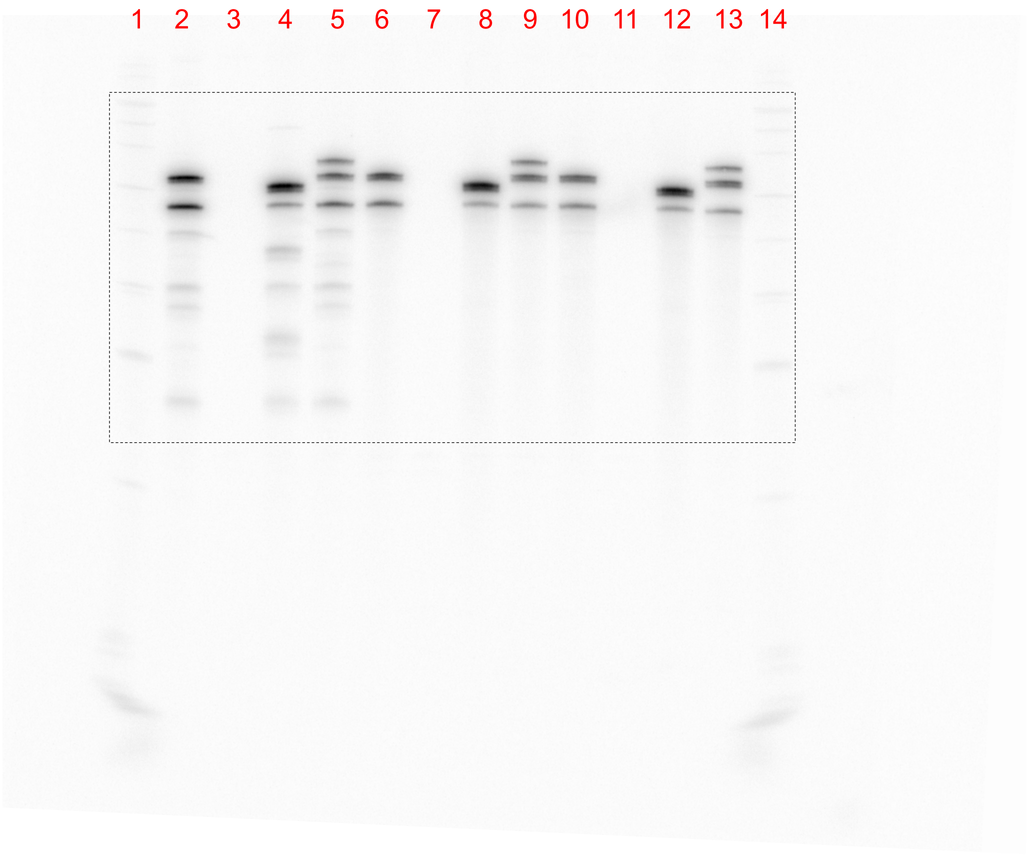

Figure S3C

5s rRNA (CD-76)

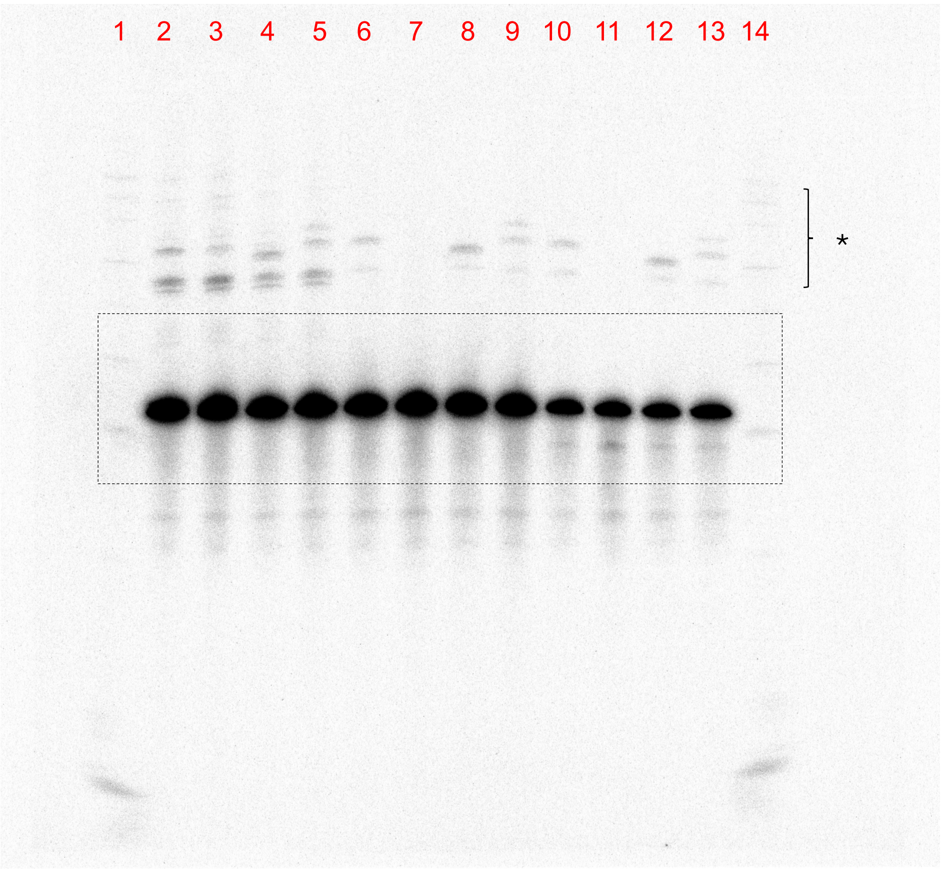

* residual signal from previous probing of ModT (FFO-67)

Figure S3G

ModT (FFO-67)

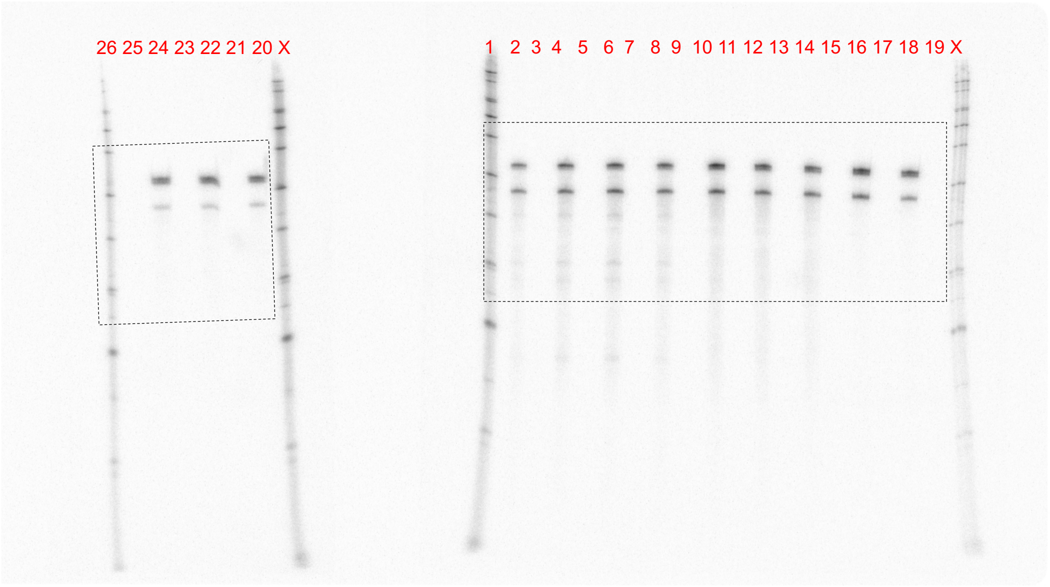

Figure S3G

tRNA-Histidine (FFO-1149)

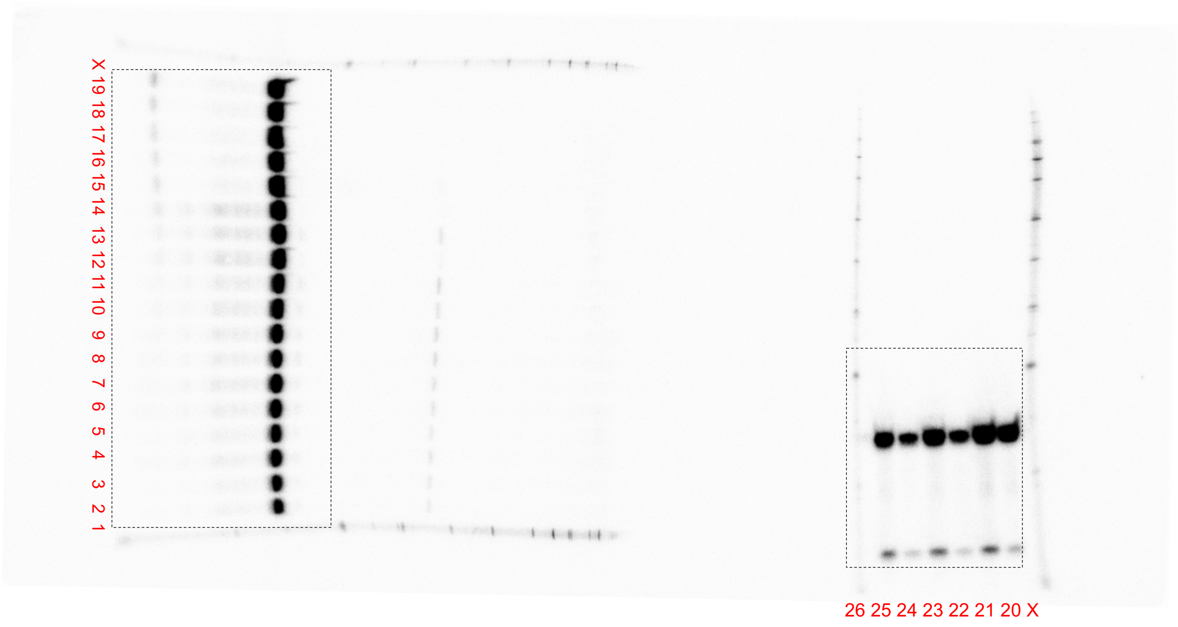

Figure S3G

tRNA-Alanine (FFO-1148)

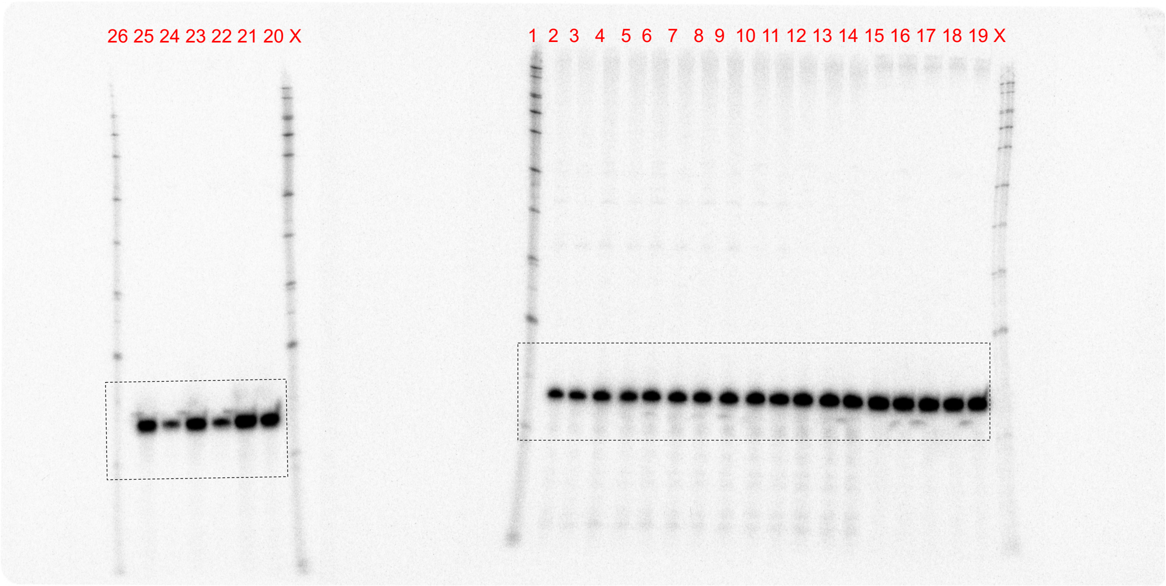

Figure S3G

5s rRNA (CD-76)

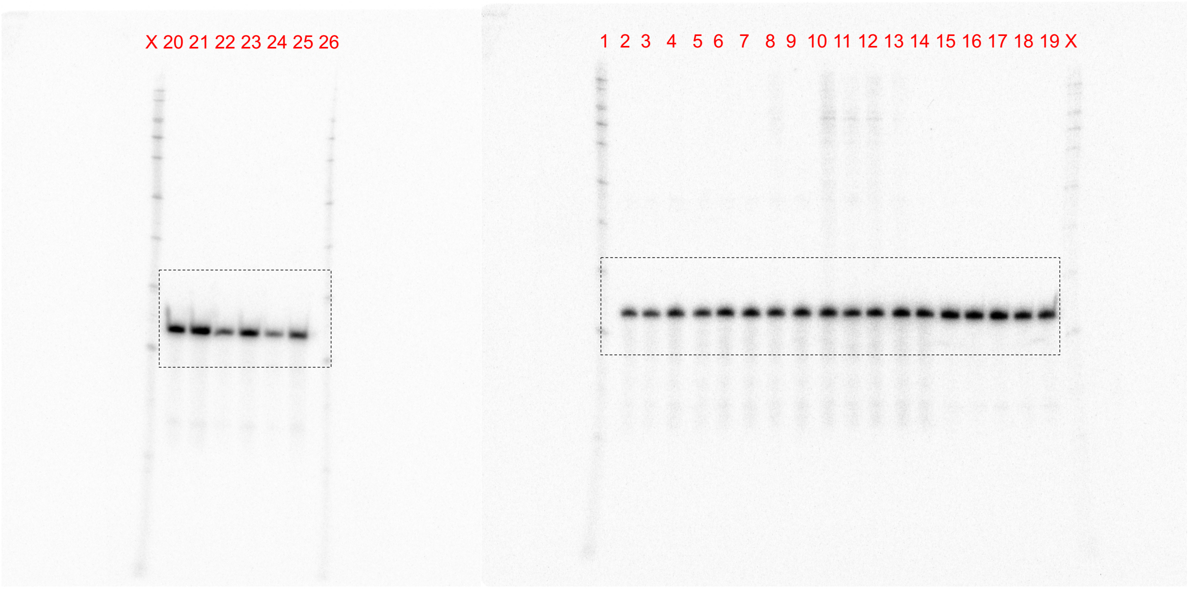

Figure S4C

ModT (FFO-67)

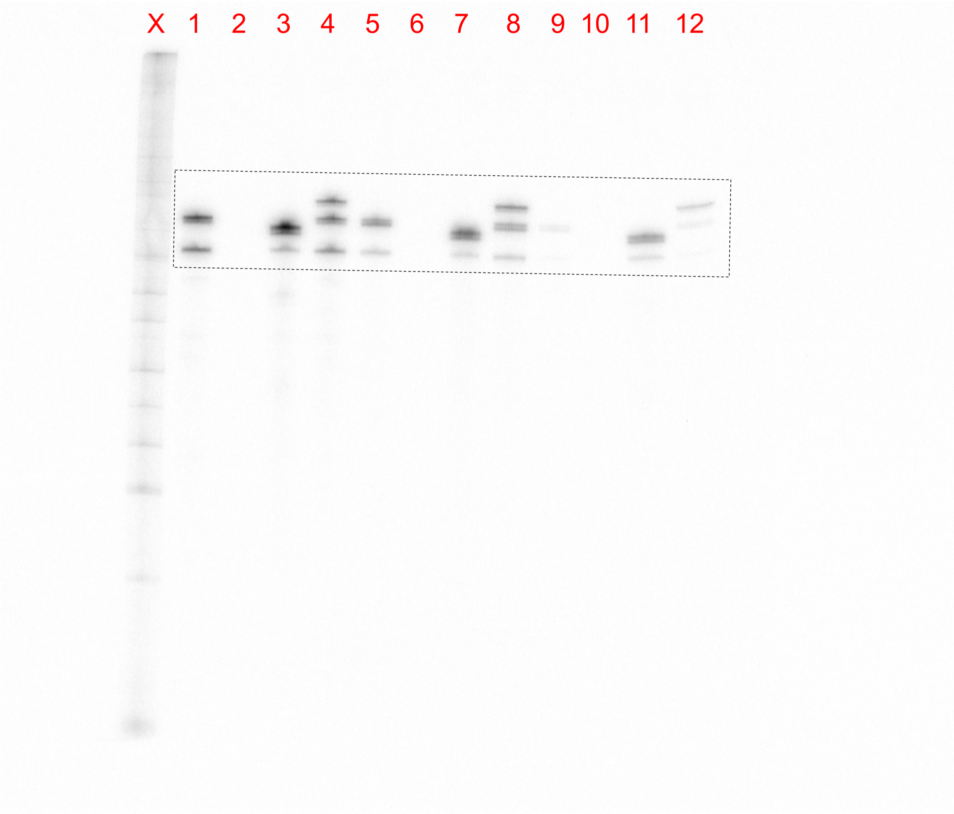

Figure S4C

5s rRNA (CD-76)

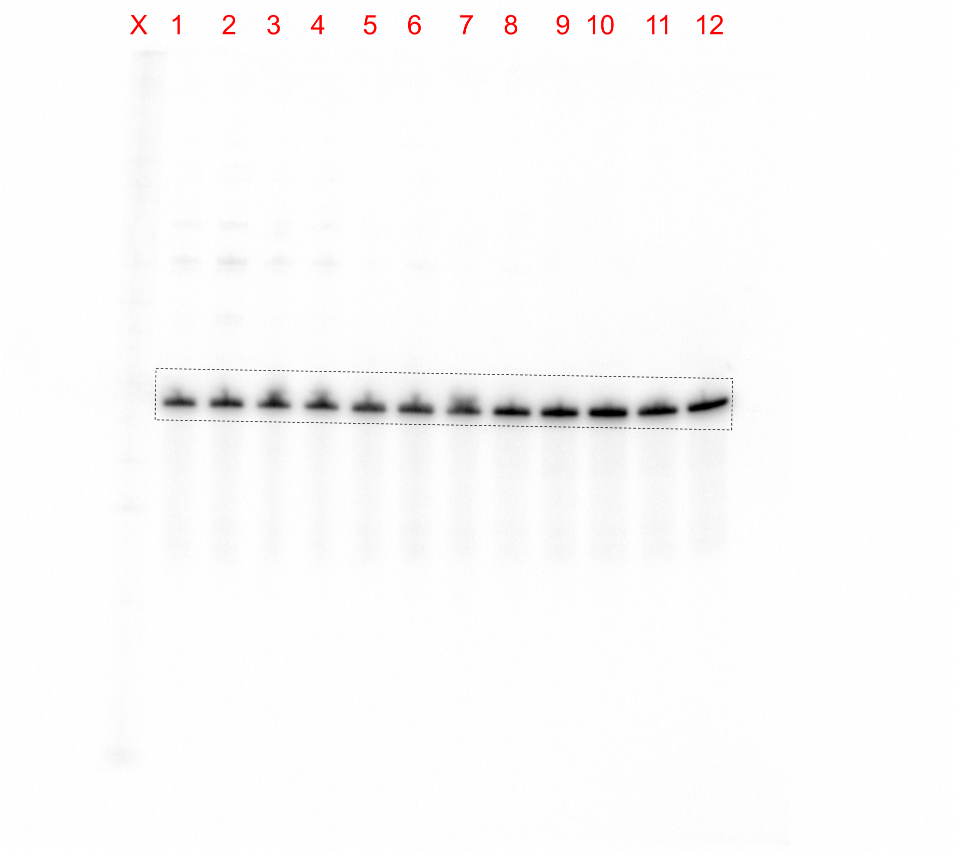

Figure S6C

ModT (FFO-1607)

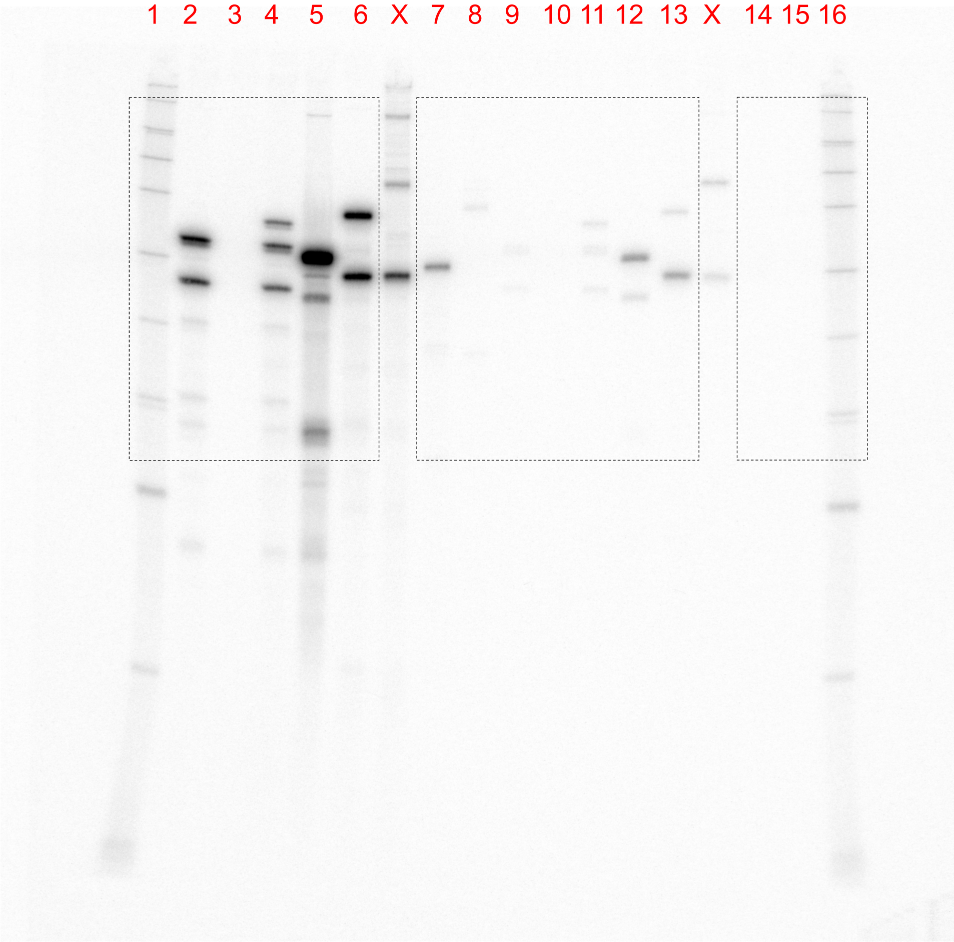

Figure S6C

ModT (FFO-1607)

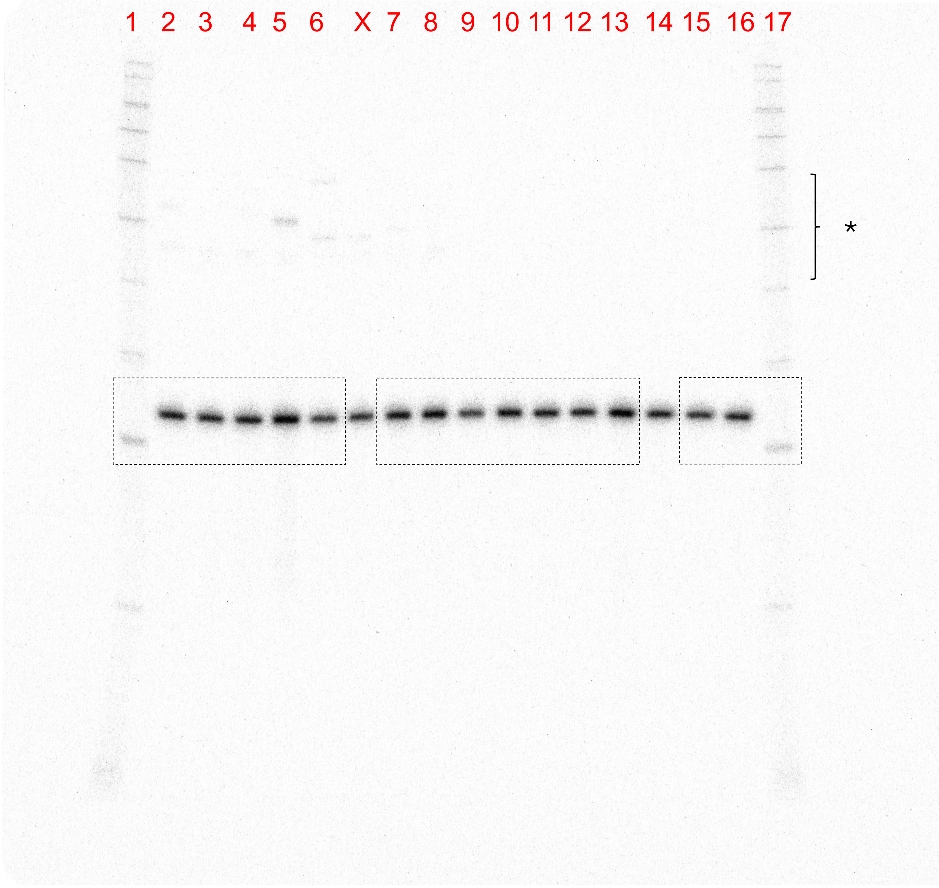

* residual signal from previous probing for ModT (FFO-1607)
